# Traditional Chinese medicine to improve immune imbalance of asthma: focus on the adjustment of gut microbiota

**DOI:** 10.3389/fmicb.2024.1409128

**Published:** 2024-10-01

**Authors:** Ke Lu, Chen Li, Jingwen Men, Bin Xu, Yang Chen, Peizheng Yan, Zhibo Gai, Qingxiang Zhang, Lu Zhang

**Affiliations:** ^1^College of Traditional Chinese Medicine, Shandong University of Traditional Chinese Medicine, Jinan, China; ^2^Innovative Institute of Chinese Medicine and Pharmacy, Shandong University of Traditional Chinese Medicine, Jinan, China; ^3^College of Pharmacy, Shandong University of Traditional Chinese Medicine, Jinan, China; ^4^Key Laboratory of Traditional Chinese Medicine Classical Theory, Ministry of Education, Shandong University of Traditional Chinese Medicine, Jinan, China

**Keywords:** asthma, effective components of traditional Chinese medicine, Chinese herbal medicine formula, short-chain fatty acids, lipopolysaccharide

## Abstract

Asthma, being the prevailing respiratory ailment globally, remains enigmatic in terms of its pathogenesis. In recent times, the advancement of traditional Chinese medicine pertaining to the intestinal microbiota has yielded a plethora of investigations, which have substantiated the potential of traditional Chinese medicine in disease prevention and treatment through modulation of the intestinal microbiota. Both animal models and clinical trials have unequivocally demonstrated the indispensable role of the intestinal microbiota in the pathogenesis of asthma. This article presents a summary of the therapeutic effects of traditional Chinese medicine in the context of regulating gut microbiota and its metabolites, thereby achieving immune regulation and inhibiting airway inflammation associated with asthma. It elucidates the mechanism by which traditional Chinese medicine modulates the gut microbiota to enhance asthma management, offering a scientific foundation for the utilization of traditional Chinese medicine in the treatment of asthma.

## 1 Introduction

Asthma is a multifaceted ailment distinguished by bronchitis, heightened airway responsiveness, and alterations in the airway structure. It stands as the prevailing chronic respiratory disorder globally. Asthma impacts approximately 300 million individuals globally, with its prevalence having increased consistently over several decades. Estimates derived from publications between 2019 and 2023 indicate that the global prevalence of asthma symptoms in children and adolescents is approximately 10%, while in adults, it ranges from 6 to 7% (Aaron et al., 2018). Research published in “The Lancet” in 2019 reveals that there are an estimated 45.7 million adult asthma patients in China, of whom approximately 70% remain undiagnosed and 95% have not received adequate treatment (Huang et al., 2019). A separate study observed trends in asthma-related hospital discharges in China from 2011 to 2020, noting a gradual increase in the number of discharged patients. Concurrently, the mortality rate associated with asthma decreased, while hospitalization expenses rose. The average expenditure per discharged patient during this period was ¥ 5,870 (Li et al., 2023). The prevalence of asthma among patients is considerable, and its chronic nature requires long-term or lifelong treatment, resulting in a substantial impact on individuals and society. The lifetime cost productivity loss constitutes approximately 60% of the overall cost associated with lifetime asthma. Given the absence of a definitive cure for asthma, current treatment primarily focuses on mitigating symptoms such as airway stenosis, asthma, and dyspnea. However, asthma significantly affects patients’ quality of life and imposes substantial economic burdens. Consequently, there exists a pressing imperative to identify a treatment modality that can mitigate the risk of asthma, enhance patient adherence, ensure safety and efficacy, and exhibit no adverse effects. Traditional Chinese medicine (TCM) holds significant importance within the realm of complementary and alternative medicine, being extensively utilized in China and neighboring nations for an extensive duration in the management and prevention of asthma. TCM exhibits numerous merits, including notable efficacy, minimal adverse reactions, and low susceptibility to drug resistance. Generally administered orally, TCM includes a variety of compounds that interact with the intestinal microbiota, producing metabolites that facilitate therapeutic outcomes.

## 2 Review of gut microbiota and asthma

### 2.1 Gut microbiota and asthma

The etiology of asthma remains incompletely understood. Research has demonstrated potential associations between asthma and immune imbalances involving Th1/Th2, Th17/Treg, as well as airway hyperresponsiveness, epithelial cell activation, mucus hypersecretion, and airway remodeling (Boonpiyathad et al., 2019). Currently, there is a heightened emphasis on investigating the microbiome and gut-lung axis in order to comprehend the etiology and progression of asthma. A longitudinal cohort study conducted on children revealed that those who exhibited susceptibility to allergic diseases within their initial year of life displayed diminished diversity in their intestinal microbial composition. Additionally, an enrichment of asthma-associated flora, namely *Anaerostipes hadrus*, *Fusicatenibacter saccharivorans*, *Eubacterium. halalii*, and *Blautia wexlerae*, was observed. The *A. hadrus*, *F. saccharivorans*, and *E. halalii* that metabolize butyric acid in SCFAs decreased significantly. The enriched species and metabolites in children with allergies were related to pathogenic activity and adverse outcomes. The role of gut microbiota in regulating human health has become the focus of research (Hoskinson et al., 2023). Healthy humans harbor a substantial microbial community comprising bacteria, fungi, viruses, and other microorganisms. Notably, the human intestine contains an estimated range of 100,000–100 billion bacteria per milliliter of lumen content (Thomson et al., 2003). The organ exhibits the highest bacterial colonization density, primarily consisting of *Bacteroidetes* (63.85%), *Firmicutes* (29.90%), *Fusobacteria* (2.98%), *Proteobacteria* (2.74%), and *Actinobacteria* (0.44%) (Hillman et al., 2017). Intestinal bacteria perform a diverse range of functions, encompassing the synthesis of vitamins, ion absorption, protection against pathogens, tissue growth, reinforcement of immune response, and food fermentation. The disruption of the intestinal microbiota will impair the integrity of the intestinal barrier and diminish the expression of occludin and zonula occludin-1 (ZO-1) tight junction proteins within the intestinal mucosa. Consequently, this will facilitate heightened intestinal permeability and the release of endotoxins into the bloodstream, thereby triggering an immune response in the lungs (Sender et al., 2016).

However, bacteria are not the sole microbial inhabitants of the gut and are insufficient to mediate all the functions attributed to the gut microbiota. Emerging evidence indicates that viruses and fungi also play crucial roles in regulating physiological functions through complex interactions with host cells during their symbiotic relationship with the human gastrointestinal tract. Despite their relatively low abundance, constituting approximately 0.1% of the intestinal microbial DNA, these microorganisms significantly contribute to the overall functionality of the gut ecosystem (Qin et al., 2010). Fungi exhibit two primary lifestyles: symbiotic and pathogenic. Symbiotic fungi can transition into pathogenic forms due to influences from the local tissue environment, host-related factors, fungal genetic and epigenetic modifications, and interactions with other microbial populations. Additionally, the bacterial microbiome significantly impacts the fungal microbiome. Interactions between fungi and bacteria, as well as between fungi and other microbial groups, can profoundly influence host health and disease dynamics (Cui et al., 2013). Intestinal fungi exhibit immunomodulatory properties within the human body. Moreover, a competitive interaction exists between the intestinal microbiota and intestinal fungi. For instance, a significant negative correlation has been observed between *Penicillium* and *Faecalibacterium*, as well as between yeast and *Lachnospiraceae*, in the intestines of healthy individuals (Nash et al., 2017). Following treatment with various antibiotics, such as cefoperazone or a combination of ampicillin, neomycin, vancomycin, and metronidazole, there is a potential for increased fungal proliferation within the intestinal tract of mice. Moreover, in instances where the immune system is compromised or antibiotics are excessively utilized, there is a heightened risk of fungal overgrowth, which may result in subsequent infection or disease (Dollive et al., 2013).

*Candida albicans* is a prevalent symbiotic microorganism in the human intestine (Kumamoto et al., 2020). However, this fungus is infrequently detected in the feces of laboratory mice, which are raised under controlled conditions that restrict their exposure to a diverse array of microorganisms. Research indicates that fungi, including *Candida albicans*, can engage with immune cells in the host’s intestinal tract via pathways involving Dectin-1, Toll-like receptor 2 (TLR2), and/or Toll-like receptor 4 (TLR4) (Li J. et al., 2018). When macrophages detect and ingest *Candida albicans*, they generate reactive oxygen species, activate MAPK and NF-kB pathways, release inflammatory cytokines, and recruit immune cells to eliminate the pathogen (Gantner et al., 2005). Conversely, *Candida albicans* can suppress the host’s inflammatory response by inducing IL-35 subunit EBI3 in M2 macrophages, thereby preventing their conversion to M1 macrophages in response to LPS (Patel, 2022). Research indicates that a single oral dose of *Candida albicans* in mice, combined with ampicillin-supplemented drinking water, results in persistent intestinal colonization by the fungus, with its levels increasing over time. Despite high levels of intestinal *Candida albicans* colonization, there is no evidence of systemic fungal transmission or adverse health effects. This suggests that such colonization can prevent systemic invasive infections by the same pathogen. Additionally, mice with *C. albicans* colonization showed significantly higher levels of Th17 cells (producing RORγt and IL-17) in their lungs compared to uncolonized control mice (Shao et al., 2019). It can prevent systemic infections by extracellular pathogens but may also increase the risk of abnormal tissue inflammation. Huffnagle and his team noted that high *Candida albicans* levels in the guts of antibiotic-treated mice worsened allergic airway disease symptoms (Noverr et al., 2004). A study treated wild-type mice with fluconazole in drinking water for 3 weeks, followed by intranasal HDM immunization 24 h after stopping the drug. Continuous fluconazole treatment worsened allergic airway inflammation (AAD). The three-week fluconazole regimen increased fungal *α* diversity, which normalized after stopping the drug. However, the total fungal 18S rDNA remained reduced 3 weeks post-treatment. In the mycobiota-free mouse model (MyF-ASF), colonization by dysfunctional fungi like A. amstelodami, *E. nigrum*, and W. sebii worsened AAD symptoms, suggesting that intestinal fungi can influence the gut-lung axis and impact peripheral immunity and disease (Li X. et al., 2018).

The quantification of virus-like particles (VLPs) in the lumen and mucosal environment of adult enteroviruses reveals approximately 10^9^–10^10^ VLPs per gram of feces or biopsy samples. Human enterovirus compositions are highly personalized, influenced by geographical location, lifestyle, diet, and age (Shkoporov and Hill, 2019). The human virus group includes eukaryotic and prokaryotic viruses (phages), with eukaryotic viruses making up less than 10%. Eukaryotic DNA viruses (e.g., herpesviruses, ring viruses, adenoviruses) are typically latent, regulating host immunity, while eukaryotic RNA viruses are uncommon in healthy individuals. Under stress from infection, pathogenic RNA viruses can emerge in the intestine, posing serious health risks through fecal-oral transmission, contaminated food/water, and human contact (Zuo et al., 2021). COVID-19, caused by the RNA virus SARS-CoV-2, primarily affects the respiratory system but also involves the gastrointestinal tract in 5–33% of patients, causing symptoms like diarrhea, nausea, and vomiting. Studies show that active SARS-CoV-2 can persist in the body even after respiratory symptoms have cleared. These findings indicate that live SARS-CoV-2 could persist in the gut, posing a fecal-oral transmission risk. Additionally, phages (bacterial viruses) significantly increased in the human intestine, making up over 90% of the enterovirus group. Phages and bacteria, along with viruses/phages and the host immune system, are fundamental to the role of human enteroviruses in health and disease. Phages, natural bacterial parasites, influence bacterial composition, metabolism, and evolution. Enteroviruses are vital for the human immune system’s development and function, and their composition and function are, in turn, regulated by interactions with gut bacteria and host immunity.

Enteroviruses can disrupt intestinal epithelial cells and alter microbial communities, while bacteriophages can influence bacterial metabolism and activity. For instance, *E. coli* infected with phage showed increased acid resistance (Veses-Garcia et al., 2015). Filamentous phages can alter bacterial traits by regulating their gene expression, enhancing the bacteria’s environmental adaptability, virulence, metabolism, biofilm structure, and toxin production (Mai-Prochnow et al., 2015). Viral infections can alter the gut microbiome by triggering immune responses. Numerous viruses and phages in the gastrointestinal mucosa influence the host’s innate and adaptive immunity (Popescu et al., 2021).

Fungi and viruses, key components of the human gut microbiota, can trigger both innate and adaptive immunity at barrier surfaces. They influence immune responses, health, and inflammatory diseases. Despite advancements, our knowledge of their interaction with the mucosal immune system remains limited. Deep sequencing revealed significant variation in fungal and viral compositions across populations, with some species common to multiple groups. The study of interactions between viruses, fungi, bacteria, and human hosts is a growing research area. A significant portion of contemporary research on the intestinal microenvironment predominantly utilizes the sequencing and analysis of 16S ribosomal RNA genes, thereby restricting the focus to bacterial populations. Consequently, there remains a pressing need for an exhaustive understanding of the viral and fungal communities within this ecosystem.

A significant intercommunication between the gut microbiota and the lungs, commonly called the lung-gut axis, has been demonstrated to impact lung immunity (Zhang et al., 2020). This axis facilitates the transportation of endotoxin, microbial metabolites, cytokines, and hormones into the bloodstream, thereby establishing a connection between the lung and the intestine. Research has demonstrated that during the initial month of an individual’s life, the prevalence of particular strains of intestinal flora, such as *Faecalibacterium*, *Lachnospira*, *Rothia*, *Bifidobacterium*, and *Akkermansia*, can influence the development of allergic asthma (Arrieta et al., 2015; Liu C. et al., 2022). A reciprocal relationship exists between the lungs and the intestines, wherein inflammation in the former impacts the gut microbiome, consequently contributing to the emergence of intestinal disorders, such as irritable bowel syndrome (Enaud et al., 2020). The decline in bacterial abundance and diversity in allergic diseases may be attributed to the ecological disruption associated with these conditions. Individuals with chronic urticaria (CU) and healthy individuals showed a significant reduction in *Akkermansia muciniphila*, *Clostridium leptum*, and *Faecalibacterium prausnitzii* (Nabizadeh et al., 2017). In a research investigation concerning the gut microbiota of children diagnosed with allergic dermatitis (AD), notable disparities were observed between children with AD and those without food allergies in terms of the presence of *Bifidobacterium breve*, *Bifidobacterium pseudocatenatum*, *Bifidobacterium adolescentis*, *Escherichia coli*, *Faecalibacterium prausnitzii*, and *Akkermansia viscophila* (Vital et al., 2015). A preliminary investigation revealed that the composition of gut microbiota exhibited variations in response to the severity of lung allergy. In comparison to mice displaying mild symptoms, mice with severe asthma exhibited a further reduction in the abundance of *Bacteroidetes* and *Firmicutes* (Vital et al., 2015).

Based on the various forms of airway inflammation resulting from an immune response, asthma can be categorized into two types: type 2 asthma and non-type 2 asthma. Individuals with type 2 asthma typically exhibit allergic reactions to common airborne allergens. The primary pathological characteristics of type 2 asthma encompass epithelial dysfunction, mucus secretion, airway smooth muscle contraction, and airway remodeling. The underlying mechanism involves the participation of helper T cells (Th) type 2 cells, type 2 innate lymphocytes, helper T follicular cells, eosinophils, mast cells, and “marker” type 2 mediators, including cytokines IL-4, IL-5, and IL-13 (Ray and Kolls, 2017). Non-type 2 asthma is correlated with various factors including air pollution, viral or bacterial infection, and obesity. This condition is characterized by the presence of Th1 and Th17 cells, neutrophil infiltration, activation of NLRP3 inflammasome, and the release of cytokines such as INF-*γ*, IL-2, IL-1β, and IL-17.A mouse model of asthma treated with bacterial-derived histamine through the intragastric route reduced IL-4, IL-5, and IL-13 levels in the lungs. In addition, this intervention reduced the type 2 inflammatory response in the OVA-sensitized asthmatic mouse model (Barcik et al., 2019). In a separate investigation, it was observed that mice induced with asthma through intranasal injection of papain exhibited heightened levels of neutrophil and IL-17 cell infiltration in the colon compared to normal mice. Similarly, human studies revealed an up-regulation of IL-17A, IL-6, and IL-8 cytokines in the peripheral blood of asthmatic patients, which was positively associated with gastrointestinal symptoms. The findings suggest that IL-17 is involved in exacerbating intestinal injury caused by asthma through the facilitation of neutrophil transport (Hong et al., 2022). The findings of a study examining the gut microbiota in children diagnosed with allergic asthma revealed that those with asthma exhibited decreased levels of *Faecalibacterium prausnitzii* and *Akkermansia muciniphila* in comparison to their healthy counterparts (Demirci et al., 2019). The expression of pili-like proteins in the outer membrane by gut microbiota has a notable impact on the development and functionality of regulatory T (Treg) cells. This stimulation of proteins results in the activation of Tregs, whereas the suppression of Tregs is associated with the excessive activation of Th2 cells in asthma (Bikov et al., 2022). Preliminary alterations in the gut microbiota, specifically those prompted by the administration of antibiotics, possess the capacity to enhance the vulnerability to asthma (Russell et al., 2012). There is a significant role for intestinal microorganisms in asthma, as they affect pulmonary inflammation by regulating the immune response (Cait et al., 2018).

### 2.2 Gut microbiota metabolites and asthma

#### 2.2.1 Lipopolysaccharide

**Figure 1:**
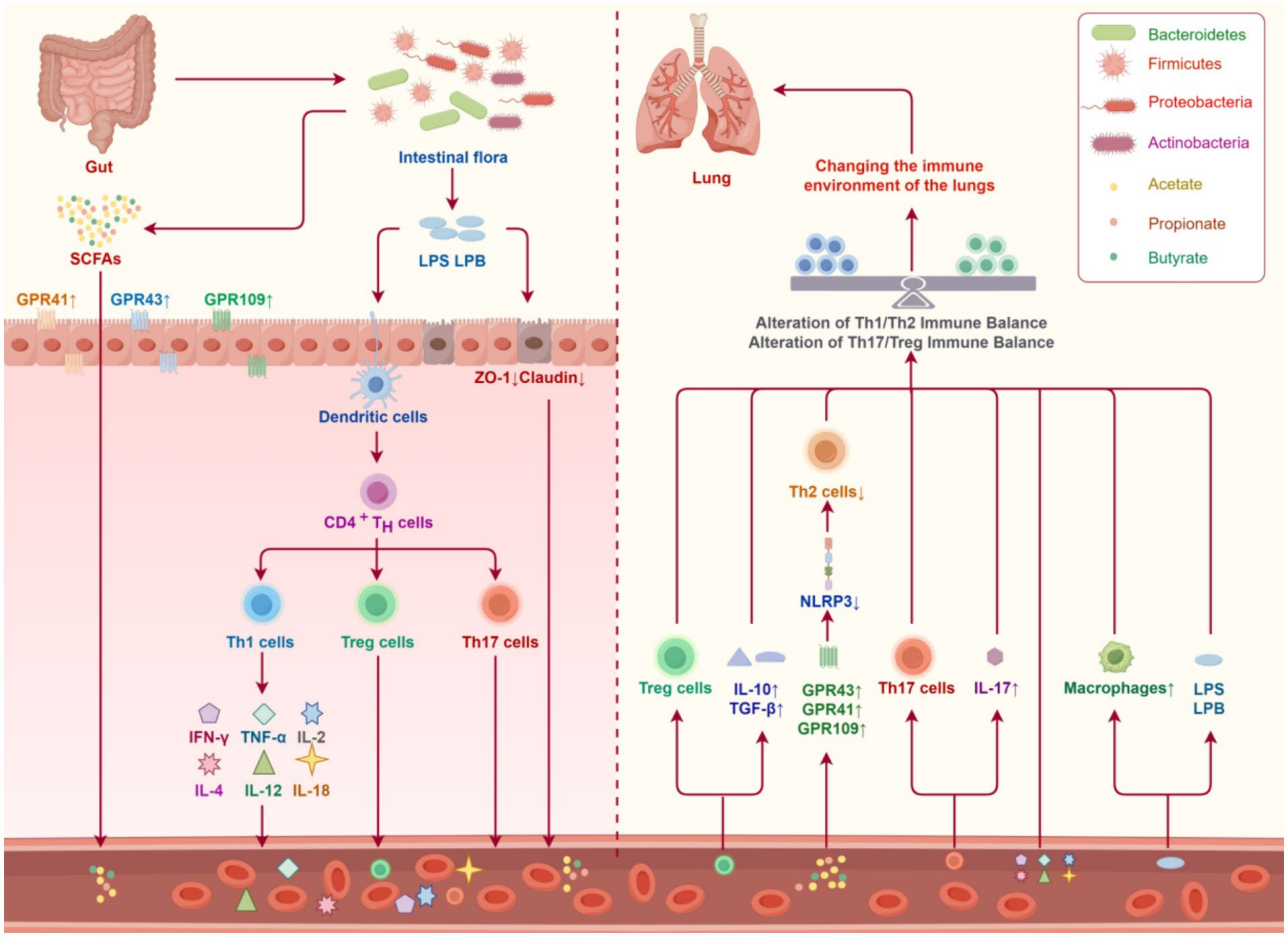
The intestinal microbiota and its metabolites influence the immunological milieu of the pulmonary system. The intestinal microbiota synthesizes short-chain fatty acids (SCFAs) and lipopolysaccharide (LPS). Acetate, propionate, and butyrate, which are constituents of SCFAs, traverse the intestinal epithelial barrier via G Protein-Coupled Receptors (GPR43, GPR41, and GPR109). These SCFAs subsequently enter the pulmonary system through the bloodstream and lymphatic circulation. In the lungs, they inhibit the activation of the NLRP3 inflammasome, thereby attenuating the function and immune response of Th2 cells. Lipopolysaccharide (LPS) binds to lipopolysaccharide binding protein (LBP) in the bloodstream, compromising the integrity of intestinal epithelial cells and increasing intestinal permeability. This process leads to a reduction in the expression of intestinal tight junction proteins, specifically ZO-1 and Claudin. Consequently, intestinal dendritic cells are stimulated, which in turn activate T cells to differentiate into Th1, Treg, and Th17 subsets. Th1 cells secrete cytokines such as IFN-*γ*, TNF-*α*, IL-2, IL-4, IL-12, and IL-18. These cells and their associated cytokines are then transported to the lungs via the circulatory system. Alter the immune equilibrium of Th1/Th2 and Th17/Treg populations within the lung, thereby influencing the pulmonary immune milieu (created by Figdraw).

Lipopolysaccharide (LPS) serves as the primary constituent of the cell wall in Gram-negative bacteria. Its status as an allergen that elicits allergic airway inflammation has garnered considerable interest. In conjunction with inhaled allergens, lipopolysaccharide derived from intestinal microorganisms may directly contribute to the initiation of asthma-related inflammation (Figure 1). Preliminary investigations have demonstrated that prolonged exposure to elevated concentrations of lipopolysaccharide can disrupt the Th1/Th2 balance, instigate asthma, and augment pulmonary inflammation (Doreswamy and Peden, 2011). A healthy intestinal microbiota maintains the homeostasis of the intestinal milieu in the human gastrointestinal tract. Probiotics exhibit the ability to counteract the metabolic endotoxemia and inflammatory response elicited by pathogenic bacteria through mechanisms such as pH modulation within the intestinal cavity, inhibition of bacterial adhesion and translocation, as well as the secretion of antibacterial substances and defensins (Mueller et al., 2015).

The symbiotic relationship between the gut microbiota and the host mucosal immune system plays a crucial role in preserving the well-being of the host, serving as the primary barrier against the infiltration of intestinal microbes. This defense mechanism encompasses various components, such as a robust mucus layer, tight junction proteins, and antimicrobial proteins, which collectively contribute to the immune response at the mucosal surface. Furthermore, the intestinal innate immune cells exhibit a remarkable ability to discern between commensal bacteria and invasive pathogens, thereby establishing tolerance toward the former and impeding their entry into the systemic circulation from the intestinal lumen (Wang C. et al., 2019). An ideal healthy microbial community should exhibit both diversity and balance. When the diversity is diminished or the microecological equilibrium is disrupted, it results in microecological imbalance, characterized by the proliferation of Gram-positive bacteria and heightened release of endotoxin. The heightened levels of endotoxin in the intestine permeate into the bloodstream due to the compromised integrity of the intestinal barrier. This endotoxemia triggers the activation of the nuclear factor-κB (NF-κB) pathway and upregulates the expression of IL-6 and TNF-*α*, thereby exacerbating inflammation associated with asthma. Symbiotic bacteria have the ability to stimulate dendritic cells (DCs) via their antigen presentation, toll-like receptor (TLR)-4 is activated, causing inflammasome overexpression of the downstream NLRP3 nucleotide-binding oligomerization domain-like receptor family (Wood et al., 2019). This phenomenon results in the polarization of macrophages toward a pro-inflammatory M1 phenotype, thereby exacerbating the inflammatory response. Additionally, it has been observed that even low concentrations of lipopolysaccharide (LPS) can stimulate T helper cells (Th2) to facilitate the promotion of allergic reactions (Tan et al., 2010).

#### 2.2.2 Short-chain fatty acids

Short-chain fatty acids (SCFAs) are a group of metabolites generated by specific symbiotic bacteria within the gut microbiota. These bacteria, such as *Faecalibacterium prausnitzii*, *Roseburia intestinalis*, and *Anaerostipes hadrus*, utilize complex carbohydrates and dietary fiber for fermentation, resulting in the production of SCFAs. The intestinal microorganisms metabolize these SCFAs, which are subsequently transported by epithelial cells through active and passive mechanisms. This transportation allows SCFAs to contribute to intestinal repair and the maintenance of intestinal homeostasis in the normal colon. Short-chain fatty acids (SCFAs) have been implicated in various aspects of asthma (Figure 1). Initially, SCFAs have been found to augment the anti-inflammatory characteristics of adaptive immune cells and exert influence over a range of physiological mechanisms. Moreover, SCFAs are involved in the modulation of T cells within the gastrointestinal tract and other tissues. In instances of pathological conditions, both acetate and propionate, whether administered individually or in conjunction, have demonstrated efficacy in mitigating inflammation by diminishing Th1/Th17 responses and elevating Treg levels (Mandaliya et al., 2021). Subsequently, the introduction of short-chain fatty acids into the system serves to reinstate the integrity of the intestinal barrier and ameliorate systemic inflammation. Acetate, predominantly synthesized by *Bifidobacterium*, plays a pivotal role in preserving the functionality of the intestinal epithelial barrier and modulating intestinal inflammation through the activation of the G protein receptor (GPR) 43 (Yoo et al., 2020). Acetate plays a significant role in the promotion of microbial monomer active IgA production through GPR43 signal transduction (Wu et al., 2017). This association can be attributed to acetate being a prominent metabolite produced by intestinal microbes, which effectively enhances IgA production in the colon and facilitates the coating of bacteria with IgA (Takeuchi et al., 2021). The induction of IgA by acetic acid is crucial for preserving the equilibrium of the intestinal microflora, while short-chain fatty acids have the potential to ameliorate endotoxemia. Propionate and butyrate were discovered to have extra inhibitory impacts on LPS-triggered NF-κB activation, while also reducing the generation of ROS and TNF-*α* by neutrophils and macrophages. These effects are achieved through the binding of propionate and butyrate to GPR41, GPR43, and GPR109A receptors, as well as through the inhibition of histone deacetylase (Cho and Shore, 2016).

In comparison to individuals without asthma, the presence of short-chain fatty acids (SCFAs) in asthma patients has been found to have a significant impact on the restructuring of gut microbiota in terms of composition and function. A study conducted in the UK examined the fecal microbiota of both healthy individuals and those with asthma, revealing a noteworthy decrease in microbial diversity among the asthmatic group. Additionally, the depletion of *Faecalibacterium prausnitzii*, *Sutterella wadsworthensis*, and *Bacteroides stercoris*, along with an increase in *Eggerthella lenta*, along with an increase in *Eggerthella lenta*, were observed. These findings suggest a potential correlation between the symptoms experienced by asthma patients and the SCFAs produced through microbial metabolism (Wang et al., 2018). A study investigated the impact of *Faecalibacterium prausnitzii* on an allergic asthma mouse model (Hu et al., 2021), both butyric acid and salicylic acid, which are produced by *Faecalibacterium prausnitzii*, exert inhibitory effects on the production of IL-8 by blocking the activation of nuclear factor (NF)-κB, thereby regulating the inflammatory process. Furthermore, the constituents of *Faecalibacterium prausnitzii* induce peripheral blood monocytes, dendritic cells, and macrophages to generate anti-inflammatory IL-10, consequently inhibiting the synthesis of pro-inflammatory cytokines such as IL-6 and IL-12 (Sokol et al., 2008). The findings revealed that asthma-induced intestinal microbial imbalance led to an increase in short-chain fatty acids (SCFAs) production, a decrease in inflammatory factors such as IL-4 and IL-5, an elevation in the proportion of Treg cells, and a mitigation of airway inflammation in mice with allergic asthma induced by house dust mite (HDM). This was achieved through the supplementation of live and dead probiotics *Faecalibacterium prausnitzii*, which primarily functioned in the production of butyric acid (Tan et al., 2023).

#### 2.2.3 Bile acids

The average bile acid pool in healthy individuals ranges from 3 to 5 g (Lanzini and Lanzarotto, 2000). Approximately 95% of this bile acid pool is actively reabsorbed in the distal ileum, while around 5% is excreted in the feces. Following the synthesis of cholesterol in the liver, primary bile acids are reabsorbed from the terminal ileum and transported back to the liver via the enterohepatic circulation (EHC). Upon returning to the liver through the portal vein, primary bile acids inhibit cholesterol biosynthesis and subsequently suppress further bile acid synthesis (Hofmann, 1999). However, a minor proportion of primary bile acids (BAs) also reach the colon. The principal intestinal bacteria implicated in bile acid metabolism include *Bacteroides*, *Clostridium*, *Lactobacillus*, *Bifidobacterium*, and *Ruminococcus*. These microorganisms facilitate the conversion of cholic acid (CA) and chenodeoxycholic acid (CDCA) into secondary bile acids through processes such as uncoupling, oxidation/epimerization, and (7-*α*-) dehydroxylation, resulting in metabolites like deoxycholic acid (DCA), ursodeoxycholic acid (UDCA), and lithocholic acid (LCA) (Ridlon et al., 2016). Secondary BAs demonstrated significant antibacterial and cytotoxic properties. Both primary and secondary BAs influence host cells by activating nuclear and plasma membrane receptors like FXR and TGR5. FXR activation by NKT cells also exhibited anti-inflammatory effects, reducing IFN-*γ* and TNF-a levels while increasing IL-10 (Fiorucci et al., 2009). FXR signaling in liver and intestinal macrophages reduces inflammation by blocking NF-κB pathways and preventing the activation of IL-1β, TNF-a, NLPR3 inflammasome, and caspase 1 (Fiorucci et al., 2018).

Bile acid helps maintain epithelial integrity by activating TGR5 and FXR receptors in intestinal cells, which boosts tight junction protein expression, enhancing barrier integrity and reducing bacterial translocation and inflammation (Verbeke et al., 2015). LCA and UDCA prevent apoptosis and reduce inflammation in intestinal epithelial cells. LCA activates PXR to enhance cell motility, while TCA and DCA regulate the Src/EGFR/ERK pathway to control cell proliferation (Terc et al., 2014). Bile acids also prevent goblet cell loss via FXR and boost Muc2 expression and mucin production independently of FXR, aiding in defense against bacterial pathogens (Gadaleta et al., 2011). Bile acid aids epithelial regeneration by targeting the TGR5 receptor in intestinal stem cells. TGR5-deficient mice, unlike wild-type mice, exhibited altered tight junction expression, increased intestinal permeability, and greater vulnerability to chemically-induced colitis, highlighting TGR5’s role in preserving the intestinal barrier (Sorrentino et al., 2020).

FXR agonists like 6-ethyl chenodeoxycholic acid (OCA) have shown anti-inflammatory and anti-fibrotic effects in lung tissues, making FXR a potential target for treating certain lung diseases (Wu et al., 2020). Research on human small airway epithelial cells and rat COPD models indicates that FXR can induce bile acid-triggered EMT in alveolar epithelial cells, potentially causing airflow limitation in COPD patients through EMT promotion in small airways (Chen et al., 2016). FXR is crucial in reducing lung inflammation and aiding lung regeneration in ALI/ARDS. It curbs the release of pro-inflammatory cytokines (IL-1*β*, TNF-*α*) and chemokines (CXCL1, MCP-1), while boosting anti-inflammatory cytokine (IL-10) levels to mitigate inflammation. ALI/ARDS triggers pro-inflammatory cytokine and chemokine release via the NF-κB signaling pathway, but FXR inhibits this pathway and also blocks the MAPK and PI3K/Akt pathways (Fei et al., 2019). In the ovalbumin-induced rat asthma model, FXR reduces airway inflammation by preventing inflammatory cell infiltration and inhibiting IL-4, IL-5, and IL-13 secretion, likely through antagonizing NFκB signaling and its target genes (Shaik et al., 2015).

#### 2.2.4 Amino acids

The gut microbiome enhances metabolite production by using amino acids from food or the host for protein synthesis and by transforming or fermenting substances. Additionally, it can synthesize several essential amino acids *de novo* (Lin et al., 2017). Proteases from both hosts and microbes break down dietary and endogenous proteins in the intestine into peptides and amino acids. These amino acids, once ingested by intestinal bacteria, can influence the gut flora by promoting beneficial bacteria and inhibiting pathogens. Intestinal bacteria can transform amino acids into bioactive metabolites, which may exhibit various biological activities and mediate the prebiotic effects of amino acids. Amino acids can benefit host health by influencing gut flora, enhancing intestinal barrier function, reducing inflammation, and improving nutrient absorption. Supplementing with amino acids boosts intestinal *β*-defensins, which have antibacterial properties, and activates the mTOR pathway by inhibiting NF-κB and MAPK inflammatory pathways (Ren et al., 2016). Combining amino acids with probiotics may create effective symbiotics. Glutamine enhances lactic acid bacteria’s acid resistance, suggesting that pairing glutamine with *Lactobacillus plantarum* could boost its viability and intestinal health benefits. Additionally, a study found that pre-supplementing *Lactobacillus plantarum* and arginine reduced liver injury and inflammation before an LPS challenge (Rishi et al., 2011).

The role of proline and its enzyme PYCR1 in allergic asthma has been highlighted by elevated levels of both in asthmatic patients. In a mouse model, deleting the Pycr1 gene reduced lung proline, airway remodeling, and epithelial-mesenchymal transition (EMT). Research indicates that PYCR1 influences EMT in airway epithelial cells through mitochondrial division, metabolic changes, and AKT/mTORC1 and WNT3a/β-catenin pathways. Inhibiting PYCR1 can mitigate HDM-induced airway inflammation and remodeling (Xu et al., 2023). Enhanced expression of L-type amino acid transporter 1 (LAT1) in activated T cells facilitates the entry of essential amino acids, crucial for their function and potentially contributing to T cell-mediated allergic inflammation. Inhibiting LAT1 can block T cell activation, offering a new treatment strategy for corticosteroid-resistant asthma (Hayashi and Kaminuma, 2022).

## 3 The present situation and advantages of traditional Chinese medicine regulating gut microbiota in the treatment of asthma

Seventy percent of China’s medical centers have TCM departments, and more and more hospitals have begun to provide distinctive TCM outpatient services (Xu and Yang, 2009; Wang et al., 2017). In the 11th revision of the International Statistical Classification of Diseases and Related Health Problems (ICD-11) by the World Health Organization (WHO) in 2019, traditional Chinese medicine was incorporated into the ICD system for the first time (Choi, 2020). This inclusion not only acknowledges the historical significance of traditional Chinese medicine in healthcare, but also signifies the increasing demand for its utilization among member countries. Traditional Chinese medicine, being an empirical medical practice, is founded upon a distinctive theoretical framework. It posits that the human body is intricately intertwined with both the natural and social surroundings, thereby asserting that individuals residing in diverse environments are subject to multifaceted influences encompassing climate, geography, living conditions, and interpersonal connections. Notably, the etiology of a given ailment may diverge according to factors such as age, gender, environment, and emotional state. It is imperative to implement personalized treatment interventions to ensure clarity for each patient and attain precise diagnosis and treatment outcomes. Simultaneously, the human body can be likened to a small universe. In traditional Chinese medicine, the liver, heart, spleen, lung, and kidney are regarded as fundamental entities that interact with six hollow organs, the physical body, and five sensory organs to establish five physiological systems. These systems work together to enhance, regulate, and maintain equilibrium within the microcosm of the human body. When external or internal factors disrupt the balance of the human environment, disease syndromes may manifest. Physicians frequently reassess the patient’s condition within a span of one to 2 weeks in order to modify the patient’s prescription and treatment until it achieves a state of stable equilibrium (Chan and Ng, 2020). The variations in the composition and quantity of human gut microbiota among individuals align with the personalized diagnostic and treatment approach of traditional Chinese medicine (Salonen et al., 2014; Yamamoto et al., 2021).

In TCM, significant emphasis is placed on the interconnection between the lung and the large intestine. According to this medical system, the lung and the large intestine are intricately linked through meridians and collaterals, exhibiting a close association in terms of physiological and pathological aspects. Consequently, pulmonary ailments frequently give rise to intestinal disorders. Furthermore, contemporary scientific investigations substantiate the notion that these two organs share a common embryonic origin. During embryonic development, the organs of the lung and intestine originate from the endoderm, a primary germ layer formed in the early stages of embryogenesis. The endoderm gives rise to various vital internal organs in adults, such as the lungs, liver, pancreas, and gastrointestinal tract. Through differentiation, distinct cell populations within the endoderm develop into specialized tissues, with some cells contributing to lung formation and others contributing to the development of the digestive system, encompassing the esophagus, stomach, small intestine, and large intestine (Green et al., 2011). Intestinal microorganisms and their metabolites exert regulatory effects on both the intestinal and pulmonary immune responses (Rastogi et al., 2022). Chinese herbal medicine, a prominent therapeutic approach in traditional Chinese medicine, has been extensively employed in the management of asthma for an extended period, surpassing the utilization of modern Western medicine (Liu et al., 2016; Lin et al., 2019). This observation underscores the considerable prospects of traditional Chinese medicine as a natural remedy with minimal adverse effects for the prevention and treatment of asthma (Che et al., 2013). The majority of traditional Chinese medicines are administered orally, whereby the active constituents of these medicines engage with the intestinal microflora upon entering the gastrointestinal tract. Consequently, traditional Chinese medicine has the capacity to elicit either stimulation or inhibition of specific microbial populations. Studies have demonstrated that TCM treatment increases the abundance of beneficial bacteria while inhibiting pathogenic bacteria’s proliferation. Moreover, TCM treatment fosters the growth of beneficial bacteria, thereby preserving a salubrious intestinal milieu (Jia et al., 2020; Yang et al., 2022). Over millennia, Chinese individuals have employed TCM as a preventive and therapeutic measure against a myriad of intricate ailments, attaining commendable therapeutic outcomes with minimal adverse effects. Consequently, TCM remains extensively utilized in contemporary practice. Due to its multifaceted nature encompassing multiple components, targets, and channels, along with its minimal toxicity and limited side effects, this approach possesses distinctive merits in addressing intricate ailments and exhibits considerable potential in asthma treatment (Zhang et al., 2022). Present investigations have indicated that the manipulation of gut microbiota as a means to manage asthma has emerged as a prominent area of focus for the development of novel therapeutics (Yuan et al., 2018).

**Figure 2:**
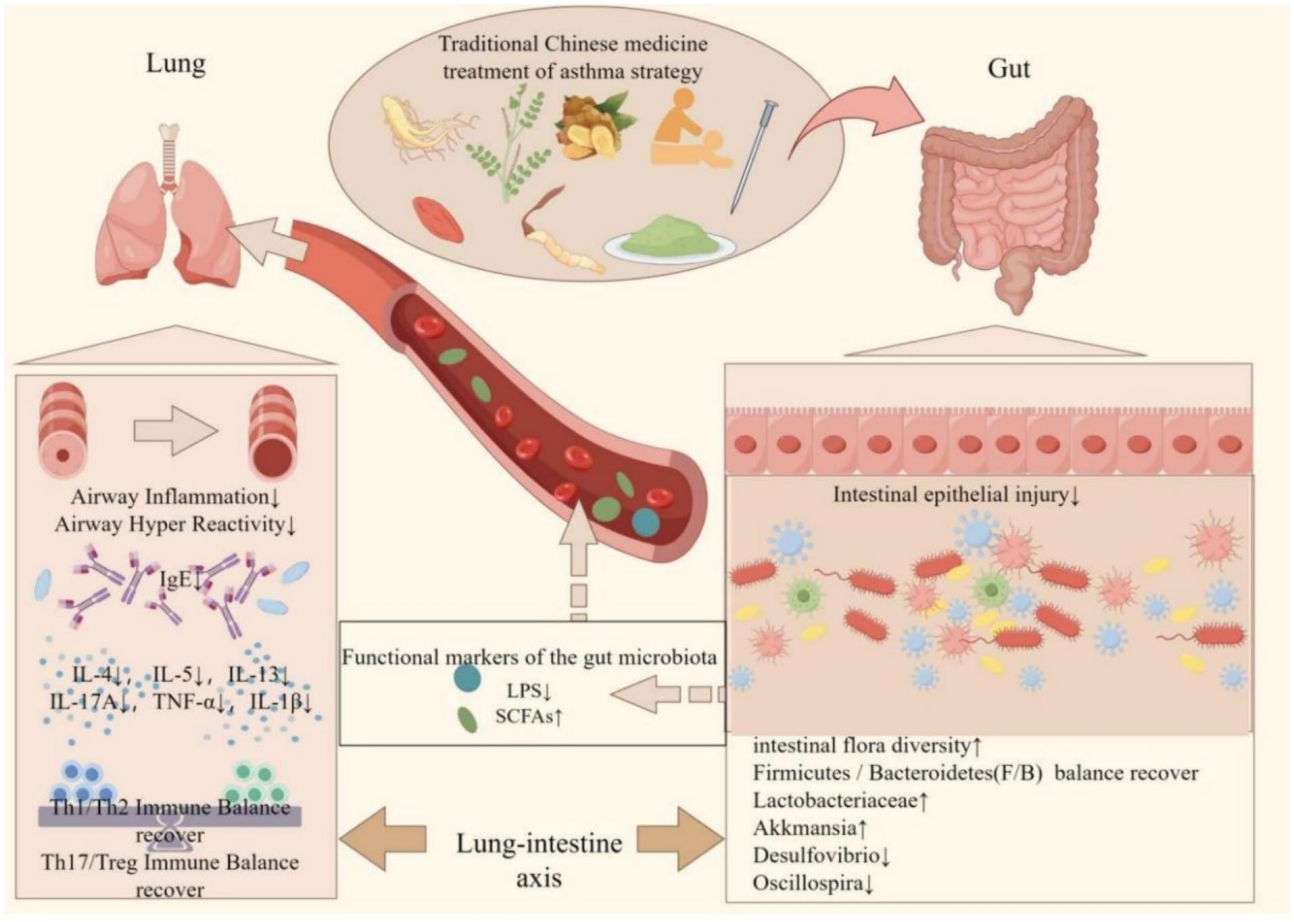
Protective mechanism of traditional Chinese medicine in improving gut microbiota in the treatment of asthma. The interaction between traditional Chinese medicine (TCM) and the intestinal microbiota may represent a potential mechanism for the treatment of asthma. Chinese herbal medicine has been shown to restore the diversity of intestinal microorganisms to varying extents, thereby re-establishing the Firmicutes/Bacteroidetes (F/B) ratio in patients with asthma. This restoration is characterized by an increase in beneficial bacteria such as Lactobacteriaceae and Akkermansia, and a decrease in harmful bacteria such as Desulfovibrio and Oscillospira. Consequently, the composition of the intestinal microbiota becomes more similar to that of healthy individuals, leading to improvements in the intestinal immune environment and the restoration of the intestinal barrier. It influences the metabolites of intestinal flora, specifically lipopolysaccharides (LPS) and short-chain fatty acids (SCFAs), from the bloodstream to the pulmonary system. This modulation results in a reduction of airway inflammation and hyperresponsiveness, a decrease in pulmonary immunoglobulin E (IgE) levels, and the restoration of immune homeostasis within the lungs (created by Figdraw).

The gut microbiota in individuals with asthma demonstrates notable correlations with risk factors for asthma, the pathogenesis of the disease, and its management and prognosis (Cho and Shore, 2016). Additionally, the integration of probiotics into the dietary regimen of asthma patients or the use of traditional Chinese medicine (TCM) can effectively regulate the gut microflora and improve intestinal function in this population (Rahman et al., 2022). TCM shows promise for both the prevention and treatment of asthma, given its status as a natural remedy with minimal negative side effects (Che et al., 2013). Consequently, TCM exhibits the ability to modulate the growth and proliferation of specific microbial groups, thereby exerting stimulatory or inhibitory effects (Figure 2). TCM has been found to have the ability to both stimulate and inhibit the growth and proliferation of specific microbial groups. Numerous research studies have demonstrated that traditional Chinese medicine (TCM) therapy has the potential to augment the composition of the intestinal microflora, promoting the proliferation of beneficial bacteria while inhibiting the excessive growth of harmful bacteria. This therapeutic approach achieves a balanced state between beneficial and pathogenic bacteria, thereby preserving a healthy intestinal environment (Jia et al., 2020; Yang et al., 2022). The therapeutic utilization of TCM facilitates the proliferation and functionality of the intestinal microbiota, which play a pivotal role in the metabolism of SCFAs (Zhang et al., 2021). Notably, the polysaccharides present in TCM pose a challenge for enzymatic degradation by human genome-encoded enzymes, yet they can be effectively broken down into absorbable SCFAs by colonic bacteria (Yue et al., 2022). Consequently, comprehending the mechanisms through which Chinese medicine modulates the composition and metabolic products of the gut microbiota becomes crucial for the treatment of asthma.

## 4 Regulating asthma gut microbiota of traditional Chinese medicine therapy

### 4.1 Chinese herbal medicine formula

Traditional Chinese medicine (TCM) is an enduring scientific discipline and cultural heritage that has been practiced for millennia. The utilization of traditional Chinese medicine prescriptions serves as the primary modality for TCM treatment, boasting an extensive historical lineage in the management of diverse ailments. Moreover, its influence has permeated beyond China’s borders, resonating in neighboring nations such as South Korea and Japan. Healthcare professionals worldwide have shown significant interest in traditional Chinese medicine prescriptions, ranging from the discovery of artemisinin in malaria treatment by Tu Youyou, the 2015 Nobel Prize laureate (Li et al., 2022), to the World Health Organization’s acknowledgment of traditional Chinese medicine in the global medical compendium. Based on the principles of TCM syndrome differentiation, a disease is categorized into various types of TCM syndrome differentiation, followed by the administration of specific Chinese medicines to address each type of syndrome differentiation and restore patients’ equilibrium (Zhang et al., 2018). The classification of traditional Chinese medicine into emperors, ministers, assistants, and messengers is determined by the primary and secondary relationship it holds with the disease. This classification effectively illustrates the distinct roles that different traditional Chinese medicines play in prescriptions. The primary focus of the “emperor” is to address the etiology of the disease and maintain homeostasis within the human body. The “minister” serves to augment the therapeutic efficacy of the “emperor.” The “assistant,” while less potent than the “emperor” and “minister,” synergistically interacts with them, thereby mitigating their potential toxicities and adverse effects. Lastly, the “messenger” facilitates the targeted delivery of all medicinal constituents to the site of pathology (Wang et al., 2012). Prescriptions frequently consist of a combination of four or more TCM. The compatibility of diverse TCM exhibits promising prospects for the treatment of intricate ailments, with fewer adverse reactions, sustained efficacy, and diminished drug-related side effects (Ma et al., 2019).

According to the principles of traditional Chinese medicine, asthma is attributed to the obstruction of the lung and airway caused by the accumulation of phlegm. The interconnection between the lung and the large intestine, as established in traditional Chinese medical theory, manifests in their close association in terms of meridian circulation, physiological processes, and pathological conditions. This perspective further elucidates the observed dysbiosis in the gut microbiota of individuals suffering from asthma (Demirci et al., 2019). Intestinal flora is consistently regulated by traditional Chinese medicine, according to numerous studies (Supplementary Table S1). Currently, the integration of gut microbiota with traditional Chinese medicine and the therapeutic application of traditional Chinese medicine targeting gut microbiota have emerged as significant breakthroughs in the field of life science. Increasingly, research on traditional Chinese medicine has underscored the pivotal importance of gut microbiota. Consequently, the investigation of gut microbiota is poised to become a crucial avenue for elucidating the intricate principles underlying traditional Chinese medicine (Li X. et al., 2021).

The study on intestinal immunity regulation found that Huaihua Powde (HHP) treatment for colitis in mice works by improving colonic barrier function and preventing microbial imbalances. Specifically, it decreased the *Bacteroidetes*/*Firmicutes* ratio, increased *Verrucomicrobia*, and inhibited *Proteobacteria* growth. Additionally, HHS administration significantly reduced the rise of *Bacteroidaceae* and the overgrowth of *Muribaculaceae*, *Lachnospiraceae*, and *Ruminococcaceae* (Liu et al., 2020). Huangqin Decoction significantly alleviated colitis in mice, reducing body weight loss, disease activity, colon shortening, tissue damage, and levels of TNF-a, IL-6, IL-1β, and COX-2 induced by DSS treatment. It also inhibited inflammatory cytokines and decreased *Desulfovibrio* and *Helicobacter*, while increasing *Lactococcus* (Yang et al., 2017). Rhubarb Peony Decoction (RPD) treatment effectively balances intestinal flora by boosting butyric acid bacteria, *Actinomycetes*, and *Firmicutes*, and restores intestinal SCFA levels. It also regulates Th17 and Treg cell proportions in mesenteric lymph nodes and modulates IL-17A and Foxp3 expression in the colon, aiding in Th17/Treg balance recovery. Additionally, RPD reduces IL-6, TNF-*α*, IFN-*γ*, IL-10, IL-17A, IL-21, and IL-22 levels in the colon while increasing Treg-related cytokines, restoring intestinal immune Th17/Treg homeostasis (Luo et al., 2019). Baitouweng Decoction alleviated colon damage by balancing Th17 and Treg cells, lowering IL-1β, IL-6, and TNF-α levels, and raising IL-10 levels. It reduced intestinal permeability in UC mice, increased tight junction proteins occludin and zonula occludens-1, and decreased p-NF-κB and p-ERK expression in the colon. It enhances the production of short-chain fatty acids like acetate, propionic acid, isobutyric acid, and isovaleric acid in the intestine, balances Th17/Treg cells, and repairs the intestinal epithelial barrier (Miao et al., 2020).

As a classic prescription for invigorating qi and strengthening the spleen, “SiJunZi decoction “is composed of *Panax ginseng* C. A. Mey. [Araliaceae, Ginseng Radix et Rhizoma], *Poria cocos* (Schw.) Wolf [Polyporaceae, Poria], *Atractylodes macrocephala* Koidz. [Asteraceae, Atractylodis Macrocephalae Rhizoma], *Glycyrrhiza uralensis* Fisch. [Leguminosae, Glycyrrhizae Radix et Rhizoma]. According to the report, administration of Sijunzi Decoction to mice with allergic asthma resulted in a notable improvement in asthma symptoms. Analysis of 16S rRNA indicated that Sijunzi Decoction promoted the proliferation of beneficial bacteria, namely *Bacteroides* and *Parabacteroides*, while exhibiting a negative correlation with airway inflammation. Enhancing the prevalence of these genera has the potential to rectify the microbial microecology imbalance, modulate the diversity of intestinal microflora, disrupt the expression levels of TLR2 and TLR7, diminish the levels of IL-4, IL-5, and IL-13 inflammatory factors, and mitigate the progression of airway inflammation in asthma (Jia et al., 2023). *Bacteroides* are widely acknowledged as advantageous in the gastrointestinal tract and play a crucial role in regulating numerous physiological processes in the human body. The increase of *Bacteroides* has the potential to induce augmented mucus degradation, thereby mitigating intestinal inflammation through the attenuation of bacterial and intestinal epithelial cell interactions (Desai et al., 2016), safeguarding the integrity of the colonic mucus barrier, and impeding the translocation of the gut microbiota metabolite LPS into the bloodstream. *Parabacteroides*, akin to *Bacteroides*, is a prevalent constituent of the intestinal microflora. They participate in the process of food digestion and metabolism, aid in the breakdown of intricate polysaccharides and cellulose, and generate advantageous metabolites (Cui et al., 2022). The intragastric administration of *Parabacteroides* in ob/ob mice resulted in the stimulation of succinic acid and secondary bile acid production within the intestine (Wang K. et al., 2019). In consequence, this stimulation enhanced intestinal permeability and alleviated inflammatory factors such as endotoxin, IL-1, and TNF present in the blood (Wu T.-R. et al., 2019).

The Guben Fangxiao Decoction, a frequently prescribed remedy for asthma treatment, is extensively employed in clinical settings. In OVA-induced asthmatic mice, the Guben Fangxiao Decoction exhibits a notable capacity to suppress the proliferation of Th17 cells, enhance Treg cells, and mitigate the advancement of asthma-related airway inflammation by modulating the immune equilibrium of Th17/Treg (Ruan et al., 2016). In relation to the intestinal microorganisms, the administration of Guben Fangxiao Decoction has been found to ameliorate the dysbiosis of the intestinal microecology and enhance the prevalence of *Firmicutes*, *Lachnospiraceae*, and *Bifidobacteriaceae*, which are predominantly responsible for the production of short-chain fatty acids (SCFAs). Additionally, there was a notable elevation in the levels of fecal acetate, propionate, and overall serum SCFAs, with a particular emphasis on the concentration of acetate. SCFAs metabolized by gut microbiota were produced in the intestine, which were circulated to the peripheral blood through the hepatic portal vein after transepithelial migration, enhancing the differentiation of Treg cells and improving systemic and local inflammatory responses (Dong et al., 2020). A separate investigation demonstrated that the administration of Qing-Fei-Shen-Shi Decoction resulted in an elevation of *Bacterium lacticum* and *Dubosie bacteria* within the intestinal microbiota, while concurrently decreasing the presence of *Leptospirillum_NK4A136_group* and *helicobacter pylori*, thereby influencing immune regulation. The administration of Qing-Fei-Shen-Shi Decoction resulted in a reduction of interleukin-4 (IL-4), interleukin-5 (IL-5), and interleukin-13 (IL-13) levels in the lungs of ovalbumin (OVA)-induced asthmatic mice, an increase in interferon-gamma (IFN-*γ*) levels, inhibition of Th2 cell differentiation, and an increase in Th1-related cytokine IFN-γ, thereby improving the Th1/Th2 imbalance. Furthermore, Qing-Fei-Shen-Shi Decoction enhanced the activity of superoxide dismutase (SOD) and glutathione peroxidase (GSH-Px) in lung tissue, reduced malondialdehyde (MDA) levels, and mitigated oxidative stress induced by asthma (Hu et al., 2023).

A separate investigation demonstrated that the Pentaherbs formula had a notable impact on mitigating airway remodeling and pulmonary inflammatory cell infiltration, as well as reducing levels of IL-4, IL-13, and IL-33, while also enhancing the Th1/Th2 ratio. Furthermore, the Pentaherbs formula was found to positively influence gut microbiota by augmenting *Bacteroidetes* abundance, diminishing *Firmicutes* abundance, and notably elevating butyrate metabolites (Tsang et al., 2018), butyrate is anticipated to hinder the activation of Th2 effector cells in the lung through its impact on the hematopoiesis of dendritic cells (Trompette et al., 2014). The Dachengqi Decoction, a traditional formula utilized for addressing constipation, demonstrates efficacy in ameliorating OVA-induced asthma and intestinal inflammation associated with asthma. This herbal remedy is observed to decrease levels of *Faecalibaculum*, a microorganism implicated in small intestinal inflammation, while concurrently promoting the growth of beneficial bacteria such as *Lactobacillus gasseri*, *Clostridium_sensu_stricto_1*, and *Senegalimassilia* (Liu Z. et al., 2023). The research on the Pingchuan formula demonstrated its potential to decrease the concentrations of IL-18, IL-6, IL-4, and Eotaxin, which are associated with type 2 asthma. This effect may be attributed to the modulation of probiotic levels, particularly *Clostridia* and *Akkermansia*, as well as alterations in serum metabolites such as acacetin and abscisic acid (Liu F. et al., 2023). There is a significant reduction in IL-4 levels in the lungs and colon after consumption of the Shaoyao-Gancao Decoction, as well as an increase in INF-γ levels. This herbal remedy also plays a role in regulating intestinal flora imbalance by decreasing the abundance of *g_Ethanoligenens* and *g_Harryflintia*, while increasing the presence of *Lachnospiraceae_NC2004_group*, *Ruminococcaceae_UCG_005*, and *Ruminococcaceae UCG-005*. SCFAs are produced by these bacteria, suggesting that Shaoyao-Gancao Decoction may alleviate asthma by modulating SCFAs and balancing Th1/Th2 responses in the lungs through the lung-gut axis (He et al., 2023).

In the murine model of allergic asthma induced by intratracheal administration of dust mite extract, dysbiosis of the intestinal microbiota was observed, characterized by a significant reduction in beneficial bacterial taxa such as *Bacteroidetes*, *Lachnospiraceae_NK4A136_group*, and *Blautia*. Following treatment with You-Gui-Wan, a reversal in the composition of the intestinal microbiota was noted, accompanied by a restoration of metabolite levels including acetylcarnitine, tryptophan, n-leucine, isoleucine, betaine, methionine, and valine (Hsu et al., 2021), and it may reduce Der p-induced inflammation and regulate Th1/ Th2 immune disorders by down-regulating TGF-*β*, IL-4, IL-5, IL-13 and inhibiting NF-κB activation and up-regulating IL-12 (Lin et al., 2012). A separate investigation yielded comparable results, indicating that the Guominkang formula decreases eosinophil count and suppresses associated cytokines, including IL-5 and IL-13. Plasma metabolic biomarkers include DL-glutamine, L-pyroglutamic acid, prostaglandin B1, and 3,4-dihydroxyhydrocinnamic acid, while *Rikenellaceae RC9*, *g_ (Clostridium)GCA900066575*, and *g_Muriculum* are identified as the principal biomarkers in gut microbiota (Zhou L. et al., 2022). The Tuo-Min-Ding-Chuan decoction inhibited TGF and IL-10 production by Treg cells in a similar study, modulation of the Th17/ Treg cell balance, and amelioration of symptoms associated with eosinophilic asthma. The plasma metabolites identified included Imidazoleacetic acid, DL-glutamine, L-pyroglutamic acid, and 2-deoxy-D-glucose, which were found to elevate levels of intestinal flora such as *Bifidobacterium* and *Corynebacterium* (Zhou F. et al., 2022). Another study indicated that Tingli Dazao Xiefei Decoction has the potential to ameliorate NO-CO metabolic disorders in both lung tissue and intestine simultaneously. This herbal remedy may modulate the levels of gas signaling molecules NO and CO, which are exchanged between the lung and intestine, leading to the regulation of Th1 cells / Th2 cells and Th17 cells/Treg cells ratios to restore immune homeostasis. Additionally, Tingli Dazao Xiefei Decoction may also increase the abundance of specific bacterial taxa, including *Lachnospiraceae_NK4A136_group*, *g_Prevotellaceae_Ga6A1*, *g_UCG_005*, *g_Intestinimonas*, and *g_Colidextribacter* (Ruan et al., 2023).

### 4.2 Chinese herbal medicine

Indigo Naturalis (IN) notably improved colitis in mice by enhancing colon tissue structure, reducing pro-inflammatory cytokines (IL-6, IL-8, TNF-a), and increasing anti-inflammatory cytokine IL-10. It also altered gut microbiota, increasing *Bacteroides*, reversing *Firmicutes* levels, decreasing *Turicibacter* (Wahab et al., 2021). Paeoniae Radix Alba reduces inflammation by decreasing Th17 cells and the IL-23/IL-17 axis. Additionally, PRADG aids in the recovery of goblet cells, epithelial cells, and colonic crypts in DSS-induced colitis mice, strengthening intestinal barriers and regulating immune response and microflora to improve colitis (Yan et al., 2022).

*Glycyrrhiza uralensis* Fisch. [Leguminosae, Glycyrrhizae Radix et Rhizoma] is frequently employed as a mediator in diverse traditional Chinese medicine concoctions. This botanical specimen primarily comprises glycyrrhizic acid and glycyrrhizin, alongside flavonoids, triterpenoids (e.g., licorice sterols and glycyrrhizic acid), polysaccharides, amino acids, among others. There are several pharmacological effects associated with these constituents, including anti-inflammatory, antioxidant, antiviral, and anti-ulcer properties (Wahab et al., 2021). A study demonstrated that *Glycyrrhiza uralensis* Fisch. can reduce goblet cell proliferation and mucus hypersecretion in the lungs and airways of asthmatic mice by inhibiting the expression of IL-13. It also controls eosinophilic inflammation around the bronchi and blood vessels by inhibiting the expression of IL-4 and IL-5 (Sun et al., 2021). A separate study has demonstrated that *Glycyrrhiza uralensis* Fisch. possesses the ability to mitigate and address diet-induced dysbiosis within the intestinal microbiota. This is achieved through the down-regulation of the *Firmicutes*/*Bacteroidetes* ratio, as well as the augmentation of acetic acid, propionic acid, butyric acid, and valeric acid production within the murine colon. Consequently, this intervention facilitates the restoration of glucose homeostasis, lipid metabolism, and epithelial barrier function (Liu F. et al., 2022).

The main components of *Bupleurum chinense* DC. [Umbelliferae, Bupleuri Radix] are mainly flavonoids, such as saikosaponin, saikgenin, quercetin, etc., and contain saikosaponin, polysaccharide, volatile oil, etc. It has been reported that found that oral administration of *Bupleurum chinense* DC. can inhibit eosinophils, goblet cells, and other inflammatory cells, improve serum total IgE, and reduce the production of Th2/Th17 cytokines by inhibiting the activation of NF-κB pathway, and regulate the imbalance of Th1/Th2 and Th17/Treg (Bui et al., 2017). Another study also found that *Bupleurum chinense* DC. can reduce the proportion of *Firmicutes*/*Bacteroidetes* by regulating the composition of intestinal flora, and adjusting and reducing the flora *Ruminococcus gnavus* related to metabolic disorders. Increase the number of *B. acidfacens* and *Akkermansia*, and improve lipid metabolism (Wu L. et al., 2021). Clinical trials have shown significant changes in serum SCFA levels in asthmatic patients after supplementation with *Lactobacillus* and the extracts of *Ocimum tenuiflorum* Burm. f. [Lamiaceae, *Ocimum Sanctum*], *Curcuma longa* L. [Zingiberaceae, Curcumae Longae Rhizoma], and *Justicia adhatoda* L. [Acanthaceae, *Adhatoda vasica* Nees]. The presence of acetic acid, propionic acid, and butyric acid exhibited notable significance in asthmatic patients who smoke, whereas propionic acid and isovaleric acid displayed significant importance in non-smokers. The administration of probiotics, specifically *Lactobacillus plantarum*, *Lactcbacillus acidophilus*, and *Lactobacillus rhamnosus*, resulted in an increase. Furthermore, the augmentation of short-chain fatty acids (SCFAs) in individuals with asthma demonstrated a potential positive correlation with improved respiratory function and reduced inflammation (Wenger et al., 2023). *Hylocereus undatus* (Haw.) Britt. et Rose [Cactaceae, Hylocere usundatus] demonstrated efficacy in ameliorating airway hyperresponsiveness and inflammation in asthmatic mice by modulating the levels of various cytokines in serum, suppressing Th2 cell differentiation, and inhibiting the expression of NF-κBp65, p38MAPK, and caspase-1 related proteins. Additionally, it led to alterations in the composition of intestinal flora, specifically reducing levels of pathogenic bacteria *Muribaculaceae* and *Alloprevotella* while increasing the abundance of *Lachnospiraceae UCG-001* and *Prevotellaceae UCG-001* (Liao et al., 2022).

Tea holds a significant role in traditional Chinese medicine, boasting a rich historical background and widespread popularity as a beverage in China. Notably, black tea and green tea are distinguished based on their fermentation processes, each believed to yield distinct effects according to traditional Chinese medicine. According to the principles of traditional Chinese medicine, green tea is commonly regarded as possessing cooling properties and is believed to possess heat detoxification, clarity-enhancing, and thirst-quenching effects. Consequently, it is frequently consumed as a traditional Chinese beverage during the summer season and finds extensive application in Chinese herbal medicine preparations. Notably, the Chuanxiong ChaTiao Powder, renowned for its efficacy in alleviating headaches, stands as a prominent prescription formulation. Black tea, classified as a fermented tea, is believed in Chinese medicine to possess warming properties that encompass both cold and warm elements. These properties are thought to enhance blood circulation and alleviate blood stasis, rendering it particularly suitable for consumption during winter. Research has indicated that both green tea and black tea exhibit the ability to modulate intestinal microecology (Li B. et al., 2022; Wu G. et al., 2022). For instance, Liubao tea, a fermented black tea cultivated in Guangxi, has been found to rectify the equilibrium between *Firmicutes* and *Bacteroidetes* within the intestinal microbiota. Additionally, it diminishes the presence of the pathogenic bacteria *Muribaculaceae*, which is linked to immune imbalance. Furthermore, Liubao tea augments the population of SCFAs-producing probiotics such as *Rikenellaceae_RC9_gut_group* and *Bifidobacterium*. Moreover, it exhibits inhibitory effects on airway hyperresponsiveness, enhances lung lesions and mucus secretion, reduces the infiltration of inflammatory cells, and displays potential therapeutic properties for asthma (Guo et al., 2023).

### 4.3 Active metabolites of traditional Chinese medicine

#### 4.3.1 Polysaccharides

Chinese medicine polysaccharides pertain to naturally occurring substances abundant in polysaccharides derived from Chinese herbal medicines. Polysaccharides are extensively present in Chinese herbal medicines and are extensively employed within the realm of traditional Chinese medicine. Their principal constituents encompass polysaccharides, comprising diverse monosaccharide units, namely glucose, xylose, and galactose. Most polysaccharides cannot be directly digested by humans due to the absence of polysaccharide hydrolase. However, intestinal flora acts as a crucial intermediary, producing enzymes that break down dietary polysaccharides into monosaccharides. These are then fermented into short-chain fatty acids in the anaerobic environment of the intestines, allowing for absorption and utilization by the human body. Oral administration of *Codonopsis pilosula* balanced cytokine expression by inhibiting IL-17A, IL-17F, IL-6, IL-22, and TNF-a, while increasing TGF-b and IL-10. This reduced the *Firmicutes*/*Bacteroidetes* ratio, promoted *Bifidobacterium* growth, and improved intestinal microbiota recovery. Additionally, it enhanced Lactobacillus and SCFA production, while inhibiting *Bacteroides*, *Desulfovibrio*, *Alistipes* spp., and *Helicobacter* (Jing et al., 2018). *Chrysanthemum morifolium* polysaccharides protect intestinal microecology by boosting anti-inflammatory factors (IL-13, IL-10, IL-4) and lowering pro-inflammatory factors (IL-6, TNF-a, IL-17, IL-23, IL-1β, IFN-*γ*). They also increase beneficial bacteria like butyrate bacillus and clostridium, decrease harmful bacteria like *Escherichia coli*, *Enterococcus*, and *Prevotella*, reverse the rise in *Bacteroides*, and reduce virulence factor expression, improved ulcerative colitis by enhancing beneficial gut flora, balancing intestinal microecology, and restoring the immune system (Tao et al., 2017). *Alhagi camelorum* Fisch polysaccharide (aAP) boosts spleen and thymus indices in mice, stimulates serum lgG and intestinal lgA antibodies, enhances intestinal cytokines, improves intestinal villi and crypt morphology, upregulates tight junction proteins (ZO-1 and Occludin), and increases intestinal immune cells (IELs and IgA^+^ cells). It activates T lymphocytes and dendritic cells in intestinal lymphoid tissues, like mesenteric lymph nodes, boosting mice immunity (Manafu et al., 2024).

Numerous studies have demonstrated the favorable therapeutic efficacy of polysaccharides derived from traditional Chinese medicine in the treatment of asthma, particularly in the mitigation of airway and systemic inflammation. *Lycium barbarum* polysaccharides exhibit the ability to diminish inflammatory markers such as TNF-*α* and IL-6 in the bronchoalveolar lavage fluid and plasma of asthmatic mice. The aforementioned outcomes were attained through the augmentation of the population of advantageous microorganisms, specifically *Lactobacillus* and *Bifidobacterium*, which play a vital role in the production of short-chain fatty acids. Additionally, this intervention facilitated the restoration of IL-4 and IL-17A levels, thereby rectifying the disrupted equilibrium between Th17 and Treg cells (Cui et al., 2020). Simultaneously, *Lycium barbarum* Polysaccharides exhibited positive effects in ameliorating chronic metabolic diseases by rectifying the dysbiosis of gut microbiota, fortifying the integrity of the intestinal barrier, mitigating systemic inflammation induced by LPS translocation across the intestinal barrier, and elevating the concentration of acetic acid (Yang M. et al., 2021; Xia et al., 2022), propionic acid, and butyric acid, suppress excessive adipose tissue accumulation, and restore levels of LDL-C, TC, TG, and other blood lipids (Gao et al., 2021; Yang Y. et al., 2021).

Inulin, alternatively referred to as inulin or fructooligosaccharides, is a naturally occurring oligosaccharide compound consisting of 2–7 fructose molecules. It is abundantly present in a diverse range of plant-based foods and Chinese botanical remedies. Traditional Chinese medicines such as *Helianthus tuberosus* L. [Asteraceae, Rhizoma et Herba Helianthi Tuberosi], *Panax notoginseng* (Burk.)F. H. Chen [Araliaceae, Notoginseng Radix], *Ligusticum chuanxiong* Hort. [Umbelliferae, Chuanxiong Rhizoma], and *Commiphora myrrha* Engl. [Buseraceae, Myrrha] contain a large amount of inulin. Inulin, functioning as a prebiotic, undergoes fermentation by advantageous intestinal bacteria, resulting in the production of short-chain fatty acids (SCFAs) like acetate, propionate, and butyrate. This process serves to ameliorate chronic inflammation in both the lungs and the entirety of the organism. Administration of inulin during pregnancy induces alterations in the maternal gut microbiota, leading to a substantial augmentation of SCFA-producing microorganisms and a subsequent mitigation of asthma-related inflammatory reactions in the offspring. The potential of the mechanism lies in the potential correlation between the production of short-chain fatty acids (SCFAs) in the mother’s inulin and the amelioration of the Th1/Th2 imbalance in the offspring’s lungs, facilitated by the placental barrier. This mechanism holds promise for the prevention of asthma (Brehm and Pfefferle, 2019; Du et al., 2020; Li L. et al., 2021; Wu Z. et al., 2022).

#### 4.3.2 Polyphenols

Polyphenols, characterized by the presence of multiple hydroxyl groups (OH), are metabolites encompassing numerous phenolic groups. These compounds are extensively present in various commonly used Chinese herbal medicines, such as *Scutellaria baicalensis* Georgi [Lamiaceae, Scutellariae Radix], *Angelica sinensis* (Oliv.) Diels [Umbelliferae, Angelicae Sinensis Radix], *Lycium barbarum* L. [Solanaceae, Lycii Fructus], etc. Polyphenols derived from Chinese medicine have been extensively employed within the realm of traditional Chinese medicine for the purpose of immune system regulation, disease prevention, and health maintenance. Curcumin treatment significantly reduced pro-inflammatory cytokines IL-1, IL-6, and CCL-2 in the colon tissue of colitis mice, while increasing anti-inflammatory cytokines IL-33 and IL-10. It also inhibited macrophage activation, regulated M1/M2 macrophage polarization, and down-regulated the protein levels of CD11b^+^, TLR4^+^ macrophages, TLR2, TLR4, MyD88, NF-κB p65, p38MAPK, and AP-1. Curcumin treats colitis in mice by balancing M1/M2 macrophage polarization and modulating TLR signaling (Kang et al., 2021).

A study conducted in 2016 demonstrated the potential of resveratrol in mitigating lung inflammation associated with obesity. This was achieved through the upregulation of phosphorylated AMPK expression in lung tissue of mice, thereby inhibiting the expression of p47 phox and the generation of reactive oxygen species (ROS). Additionally, the study observed an elevation in superoxide dismutase (SOD) levels, ultimately alleviating obesity-related lung inflammation (André et al., 2016).

Curcumin is one of the main active metabolites in *Curcuma longa* L. Curcuma longa L., a natural yellow pigment, exhibits a diverse range of pharmacological activities and health benefits. A recent study has demonstrated the efficacy of curcumin in mitigating the structural alterations in intestinal microbiota induced by a high-fat diet. Specifically, curcumin was found to down-regulate the *Firmicutes*/*Bacteroidetes* (F/B) ratio and up-regulate the abundance of *Lactobacillus*, *Lactococcus*, and *Bacteroides*. Notably, *Lactobacillus* and *Lactococcus* are classified under *Lactic Acid Bacteria*, which are capable of producing lactic acid through fermentation. This lactic acid can subsequently undergo conversion into SCFAs by other gut microbiota. Therefore, *Lactobacillus* and *Lactococcus* may indirectly promote the production of SCFAs. *Bacteroides* have rich cellulose degradation ability and can produce SCFAs by fermenting dietary fiber (Scazzocchio et al., 2020). An OVA-induced asthmatic mouse model demonstrates that tetrahydrocurcumin reduces eosinophil infiltration and mucus production within the pulmonary tissue after the double bond of curcumin is reduced. Additionally, it modulates the levels of Th2 and Th17 cells in the pulmonary tissue, thereby ameliorating the Th1/Th2 and Th17/Treg imbalances observed in asthmatic mice (Wu L. et al., 2021).

Tea polyphenols, identified as the principal constituents of tea, possess prebiotic properties that facilitate the regulation of intestinal microecology and the production of short-chain fatty acids (Zhao and Zhang, 2020). Furthermore, these polyphenols exhibit the ability to impede metabolic disorders. Research findings indicate that Epigallocatechin-3-gallate (EGCG) has the capacity to decrease the levels of inducible Nitric Oxide Synthase (iNOS) and nitric oxide metabolites (NOx), as well as reactive oxygen species (ROS) in lung tissue. Additionally, EGCG has been observed to reduce the levels of superoxide dismutase (SOD) and inflammatory factors such as TNF-*α*, IL-4, and IL-5 in the bronchoalveolar lavage fluid (BALF) of obese asthmatic mice. Consequently, these effects contribute to the amelioration of obesity-related asthma (André et al., 2018). Epigallocatechin-3-gallate (EGCG) possesses potent antioxidant properties that enable the efficient elimination of reactive oxygen species (ROS). Moreover, EGCG exerts various beneficial effects, including anti-inflammatory, anti-fibrotic, pro-apoptotic, anti-tumor, and metabolic actions, through the regulation of multiple intracellular signaling cascades. Pretreatment with EGCG mitigates lung injury and suppresses inflammatory cytokines in lipopolysaccharide (LPS)-induced lung inflammation (Wang et al., 2021). Pre-administration of EGCG prior to ovalbumin (OVA) exposure exhibits a potential to mitigate epithelial-mesenchymal transition (EMT), attenuate the recruitment of inflammatory factors, alleviate bronchial contraction, decrease the levels of total white blood cells, macrophages, eosinophils, and neutrophils, and hinder airway remodeling (Yang et al., 2018). In mice with ova-induced bronchial asthma, the administration of EGCG via tail vein injection demonstrates a notable amelioration of asthma symptoms, along with a reduction in lung infiltration of inflammatory cells and levels of inflammatory factors IL-2, IL-6, and TNF-α. Furthermore, EGCG exhibits an elevation in IL-10 levels, a decrease in the proportion of Th-17 cells, and an increase in the proportion of Treg cells through its influence on the TGF-β1 signaling pathway (Shan et al., 2018). Research has demonstrated that EGCG has the ability to alter the composition of gut microbiota in the human gastrointestinal tract. Specifically, the presence of *Bacteroides* and *Lachnoclostridium* in samples treated with EGCG is notably higher compared to control samples, and can be readily metabolized by human intestinal microorganisms. The primary microbial metabolites resulting from sequential ester hydrolysis, C-ring opening, A-ring fission, dehydroxylation, and aliphatic chain shortening processes are phenylcarboxylic acids. These interactions play a role in the advantageous effects of EGCG within the human body (Liu et al., 2020).

#### 4.3.3 Flavonoids

Flavonoids, classified as polyphenols, are naturally occurring metabolites abundantly present in various plant sources including flowers, fruits, vegetables, tea, and other plant components. These compounds hold significant importance as active metabolites within numerous traditional Chinese medicinal herbs, such as *Scutellaria baicalensis* Georgi, *Bupleurum chinense* DC., *Sophora flavescens* Ait. [Leguminosae, Sophorae Flavescentis Radix], *Citrus aurantium L.* [Rutaceae, Fructus Aurantii Immaturus], and so on. traditional Chinese medicine flavonoids are commonly acquired through water extraction, ethanol extraction, or supercritical fluid extraction. Flavonoid glycosides, which have glycosidic bonds, are less pharmacologically active and poorly absorbed in the intestine due to water-soluble sugars, leading to low bioavailability. Recent studies indicate that intestinal flora converts most flavonoids into simple phenolic acids through various reactions, enhancing their absorption and bioavailability. Puerarin, a natural flavonoid, alleviates ulcerative colitis in mice by reducing inflammation, balancing cytokines, maintaining intestinal barrier integrity, restoring immune function, inhibiting NF-κB and NLRP3 inflammasome activation, and reducing oxidative stress. It lowers pro-inflammatory cytokines (IL-1*β*, IL-6, TNF-a) and increases anti-inflammatory IL-10, thus improving disease symptoms (Wu M. et al., 2019). Baicalin reduced intestinal mucosal damage and lowered ZO-1, Occludin, and MUC2 expression. It improved colorectal inflammation by decreasing ROS and MDA levels while increasing GSH and SOD levels. This effect may be due to the regulation of Th17/Treg balance and increased intestinal microflora and SCFA, protecting rats from ulcerative colitis (Zhu L. et al., 2020).

These flavonoids are extensively employed in traditional Chinese medicine to ameliorate inflammatory ailments, with a particular focus on investigating their efficacy in alleviating chronic asthma-related inflammation. Numerous studies have demonstrated that quercetin (Park et al., 2009; Tanaka et al., 2020), for instance, rectifies the Th1/Th2 imbalance by modulating the production of Th1/Th2 cytokines, while also diminishing the expression of FcεRI (Fc epsilon RI) in BMMC (Bone Marrow Mononuclear Cell) and MC/9 epithelioid cells of asthmatic model mice, thereby enhancing allergic response (Alam et al., 2022). Quercetin significantly contributes to the regulation of gut microbiota through its ability to enhance the intestinal microenvironment, decrease the *Firmicutes*/*Bacteroidetes* ratio, augment the population of beneficial bacteria like *Akkermansia* and *Flavobacterium*, elevate the levels of SCFAs, and inhibit abnormal glucose metabolism-induced aberrant activation of TLR-4/NF-B signaling (Wu et al., 2024).

A flavonoid metabolite isolated from *Scutellaria baicalensis* Georgi was administered intragastrically to mice with asthma, the inhibition of STAT3 in lung tissue, the promotion of FOXP3 in lung tissue, the reduction in serum IgE, IL-17 A, and IL-6 secretion in BALF, the promotion of IL-10 secretion, and the improvement of Th17/Treg immune imbalance were observed (Xu et al., 2017). Simultaneously, it was observed that baicalin augmented the prevalence of *Ruminococcacee_UCG-014*, *unclassified_f_Ruminococcaceae*, and *Clostridiales_vadinBB60_group* in the chicken intestinal microbiota. Conversely, it diminished the prevalence of *Lachnospiraceae*, *Intestinimonas*, *Blautia*, *Escherichia_Shigela*, and *Pygmaiobacter*. Additionally, baicalin facilitated the generation of short-chain fatty acids (SCFAs) within the intestines and ameliorated lung inflammation induced by avian pathogenic *Escherichia coli* (Peng et al., 2021).

#### 4.3.4 Terpenes

Terpenoids are widely found in many Chinese herbal medicines, which are metabolites composed of isoprene skeletons. The intestinal microbiota possess the capability to produce β-glucosidase, an enzyme that catalyzes the hydrolysis of iridoids into aglycones. Subsequently, the hemiacetal moiety within the aglycone undergoes partial cleavage. Concurrently, ammonia generated through the nitrogen metabolism of intestinal bacteria engages in an addition reaction with the aldehyde group. This series of biochemical transformations ultimately results in the formation of nitrogen-containing compounds. This traditional Chinese medicine often has a special aroma, such as *Panax ginseng* C. A. Mey., *Zingiber officinale* Rosc.[Zingiberaceae, Zingiberis Rhizoma], etc. *Panax ginseng* C. A. Mey. harbors a diverse array of ginsenoside metabolites, which are considered quintessential terpenoids in traditional Chinese medicine. These metabolites exhibit a wide range of pharmacological effects, encompassing immune enhancement, anti-fatigue properties, and cognitive function improvement. Ginsenosides possess the capacity to augment the body’s adaptability and resilience against diverse stressors, thereby promoting physical vigor and retarding the aging process. Research has demonstrated that ginsenosides have the ability to enhance the abundance of *Akkermansia*, a beneficial bacteria, regulate the metabolism of short-chain fatty acids (SCFAs), and ameliorate damage to the intestinal barrier by restoring the *Firmicutes*/*Bacteroidetes* ratio, thereby reducing the circulation of lipopolysaccharides (LPS) derived from gut microbiota metabolites (Zou et al., 2022). In a mouse model of BALB/c asthma induced by ovalbumin (OVA), ginsenosides have been observed to decrease the population of inflammatory cells such as IL-4, IL-5, and IL-13, inhibit the activation of NF-κB/MAPK, and mitigate allergic airway inflammation (Chen et al., 2015, 2021; Li L. et al., 2021).

## 5 Discussion

Low-grade chronic inflammation is associated with a dysbiosis of gut microbiota, characterized by a decrease in the relative abundance of *Firmicutes* and *Bacteroidetes*. The symbiotic equilibrium of the gut flora plays a crucial role in preserving the integrity of the intestinal barrier and regulating immune responses. By reducing the proportion of these phyla, we may be compromising the integrity of the intestinal barrier and triggering abnormal immune responses, consequently elevating the susceptibility to inflammation. Research has demonstrated a correlation between asthma and a decrease in Firmicutes and Bacteroidetes abundance. Additionally, our research revealed an elevation in the *Firmicutes*/*Bacteroidetes* ratio within the intestinal microbiota of mice with bronchial asthma. Remarkably, this imbalance was rectified through traditional Chinese medicine interventions. This alteration could potentially be attributed to the impact of dietary fiber on the gastrointestinal microflora composition. Many traditional Chinese medicines contain significant amounts of fermentable dietary fiber, such as *Poria cocos* (Schw.) Wolf, *Allium tuberosum* Rottl. Ex Spreng., and *Glycyrrhiza uralensis* Fisch., which can influence the balance between *Firmicutes* and *Bacteroidetes*. This ratio directly influences the metabolic processing of fiber by intestinal microbial communities and leads to an elevation in circulating short-chain fatty acid concentrations (Trompette et al., 2014). Nevertheless, it is important to note that not all investigations corroborate this assertion, Zheng’s study demonstrated that *Allium tuberosum* Rottler ex Sprengle exerts regulatory effects on the gut microbiota, specifically by augmenting the population of *Firmicutes* (Zheng et al., 2021). The implementation of Chinese medicine therapy led to an increase in the prevalence of beneficial bacteria, specifically *Akkermansia* (Song et al., 2023) and *Lacticacidbacteria* (Hu et al., 2023), which play a role in the metabolism of short-chain fatty acids. This intervention resulted in a decrease in the presence of inflammation-related microorganisms, as *Desulfovibrio* (Ai et al., 2022; Wang et al., 2022) and *Oscillospira* (Wei et al., 2020; Shen et al., 2021). Simultaneously, Chinese medicine therapy has the potential to decrease serum LPS levels through the restoration of intestinal mucosal integrity and the reduction of lipopolysaccharide (LPS) co-producing bacterial populations. This regulation of gut microbiota and its metabolites, including SCFAs and LPS, leads to an improvement in the imbalance of Th1/Th2 and Th17/Treg cells in lung tissue. Currently, the predominant focus of research in traditional Chinese medicine lies in the regulation of Th1/Th2 immune imbalance and gut microbiota. This is achieved through the inhibition of Th2 cell differentiation by suppressing the production of IL-4, IL-5, and IL-13. Modulation of eosinophil inflammatory response may be attributed to inhibition of activation of NF-κB pathway (Bui et al., 2017; Liao et al., 2022; Wu et al., 2024). In cases of asthma characterized by an imbalance in Th17/Treg immune response, Chinese medicine has been shown to impact eosinophilic asthma by reducing levels of cytokines such as IL-17A, INF-*γ*, TGF-*β*, and ROR-γ. Additionally, Chinese medicine has been found to have a significant effect on probiotics such as the *Rikenellaceae_RC9_gut_group* and *g_Bifidobacterium*, and may also influence the production of metabolites including Imidazoleacetic acid, DL-glutamine, 2-deoxy-D-glucose, and L-pyroglutamic acid by promoting the growth of beneficial bacteria to modulate the Th17/Treg immune response (Ruan et al., 2016). Furthermore, in the external treatment of traditional Chinese medicine acupuncture and massage therapies have also demonstrated efficacy in modulating gut microbiota and ameliorating immune imbalance in asthma. In the context of asthma diseases, acupuncture and massage prescriptions primarily involve the utilization of BL13 Feishu, BL23 Shenshu, ST36 Zusanli, BL20 Pishu, DU14 Dazhui, EX-B1 Dingchuan, RN17 Danzhong, and spinal massage. These interventions have exhibited favorable outcomes in terms of enhancing lung function and alleviating asthma symptoms (Jiang et al., 2019). Zhu made substantial enhancements to the richness and diversity of the intestinal microbiota by employing traditional Chinese medicine massage techniques. The addition of beneficial bacteria that produce SCFAs, such as *Bacteroidales_S24-7_group*, *Ruminococcaceae*, and *Roseburia*, enhanced the production of IFN-γ and IL-10, and weakened Th2 immune imbalance and lung inflammation (Zhu Y. et al., 2020).

The management of intricate diseases through traditional Chinese medicine frequently encompasses multiple systems and extensively modulates the regulation of gut microbiota. We boldly hypothesized that Chinese medicine could potentially treat diseases, particularly those linked to inflammation, by improving the gut microbiota’s dominant bacteria and metabolites. This proposition offers a plausible rationale for the application of traditional Chinese medicine in clinical settings. In the context of a particular ailment, including the identical patient, diverse practitioners of traditional Chinese medicine may employ distinct treatment approaches and prescriptions, yet still attain comparable or equivalent therapeutic outcomes. The underlying mechanism potentially involves the modulation of gut microbiota and its metabolites. Asthma-related risk factors, such as allergens, respiratory inflammation, and immune imbalances, appear to be interconnected with imbalances in the gut microbiota. The dysbiosis of the microbiota has the potential to exert a substantial impact on the progression and prognosis of asthma through its influence on the immune inflammatory response. Consequently, there is a promising prospect of developing microbiome-centered interventions to enhance the prognostic outcomes for affected individuals.

In spite of the considerable potential of traditional Chinese medicine to treat asthma, most existing studies examine the effect of traditional Chinese medicine on the regulation of gut microbiota and its metabolites in asthma models. However, it is important to note that these studies merely establish a correlation rather than a causal relationship, and they lack practical verification in terms of both forward and reverse causality. Furthermore, the constituents of Chinese herbal medicines and formulations exhibit a high degree of complexity, and it should be noted that not all bioactive constituents can undergo transformation by the gut microbiota. Consequently, additional investigation is required to fully comprehend the interplay between the bioactive constituents of Chinese herbal medicines and their potential impact on asthma.

## 6 Conclusion and foresight

To explore the potential mechanisms by which TCM therapy could improve asthma, we analyzed the gut microbiome in this study. The results of our study indicate that the administration of traditional Chinese medicine influences the diversity, structure, and composition of the intestinal microbiota as well as its associated metabolites. Specifically, (1) the application of Chinese medicine treatment demonstrated a capacity to enhance the growth of advantageous bacteria, including *Lactic Acid Bacteria*, *Bifidobacterium*, and *Akkermansia*, while concurrently suppressing the prevalence of detrimental bacteria, such as *Desulfurization vibrio* and *Oscillospira*. This therapeutic intervention also exhibited the ability to reinstate the equilibrium of the intestinal microecology, thereby ameliorating symptoms associated with asthma. (2) The treatment of traditional Chinese medicine has the ability to modulate the metabolism of intestinal microorganisms, resulting in an increase in the concentration of short-chain fatty acids (SCFAs) and inhibition of the translocation of metabolic endotoxin LPS into the bloodstream. (3) The intestinal microbiota has the capability to convert botanical constituents into secondary metabolites that possess potent pharmacological properties. For instance, polysaccharide components can be converted into SCFAs like propionic acid and butyric acid. These SCFAs exhibit immunomodulatory effects on the human body, thereby promoting host immune and metabolic homeostasis. Consequently, advocating for the integration of traditional Chinese medicine treatment as an alternative approach for the prevention and management of asthma patients, as well as exploring the potential of natural plant active metabolites in relation to the efficacy of asthma intervention through the gut microbiota, holds significant research potential. This emerging avenue represents a novel direction in the field of asthma prevention and treatment.

Regulating gut microbiota and its metabolites, however, still poses many challenges. In the first place, existing sequencing technology is still unable to provide a comprehensive evaluation of gut microbiota, and some species may be missed during the detection process (Tessler et al., 2017). Research has indicated that caution should be exercised in assigning excessive importance to the role of intestinal microflora, as the limitations of current technologies may potentially result in erroneous conclusions (Sorbara and Pamer, 2022). Moreover, numerous deficiencies exist in the traditional Chinese medicine approach to treating asthma via manipulation of gut microbiota. Notably, a dearth of studies investigating the asthma disease model represents a significant issue, alongside a lack of subsequent verification experiments. As a result of the lack of standardized protocols for developing and assessing asthma models, the lack of high-quality, multi-center studies with large sample sizes is due to the limited size and scope of clinical samples. Consequently, the specific Chinese herbal medicine constituents within traditional Chinese medicine formulas that exert a pivotal pharmacological influence on the modulation of intestinal microbiota remain uncertain. Hence, Further assessments are necessary to evaluate the effects of different Chinese medicine treatment modalities on the intestinal microbiota of asthma patients, in order to establish a quantitative framework for assessing the efficacy of Chinese medicine. Additionally, efforts should be made to enhance the safety and effectiveness of Chinese medicine treatment evaluations. To develop a precise treatment approach, it is essential to delve deeper into the active constituents of Chinese herbal medicine, identify the specific components associated with relevant intestinal microbiota and their metabolites, and conduct comprehensive experimental investigations for thorough validation. (1) The development of novel gut microbiome sequencing techniques is imperative in order to effectively characterize the gut microbiome and accurately identify the regulatory active components present in natural plants. (2) There is a need for standardized and unified stable models in animal studies of asthma to accurately assess the true impact of drugs. (3) The mechanism by which Chinese medicine treatment regulates gut microbiota should be validated in asthma models involving rodents and large mammals. (4) Rigorous clinical trials are urgently needed to examine the effectiveness of Chinese herbal medicine in the treatment of asthma. Furthermore, it is imperative to establish a quantitative framework for evaluating the effectiveness of traditional Chinese medicine, thereby facilitating its safe and efficient utilization.
