# Supplementary material for: Traditional Chinese medicine to improve immune imbalance of asthma: focus on the adjustment of gut microbiota
Source: Front Microbiol. 2024 Oct 1;15:1409128. doi: 10.3389/fmicb.2024.1409128 (PMC11473343; doi:10.3389/fmicb.2024.1409128)
Supplement: Supplementary file 1 [file Table_1.docx]

**Table 1.** Chinese medicine regulates gut microbiota and its metabolites to treat asthma

| Type | TCM | Parts/components | Main active components | Chemical structure | Molding method | Gut microbiota changes | Functional markers of the gut microbiota | Clinical manifestation | Results | reference documentation |
| --- | --- | --- | --- | --- | --- | --- | --- | --- | --- | --- |
| SCFAs | Gu-Ben-Fang-Xiao Decoction | *Astragalus membranaceus* (Fisch.) Bunge [Leguminosae, Astragali Radix ], Codonopsis pilosula (Franch.) Nannf. [Campanulaceae, Codonopsis Radix ], Atractylodes lancea( Thunb.)DC. [Compositae, Rhizoma Atractylodis ], Poria cocos (Schw.) Wolf. [Polyporaceae, Poria ], Ostrea gigas Thunberg. [ostreae, Concha Ostreae], Cryptotympana pustulata Fabricius [Cicadidae, Cicadae Periostracum ], Citrus reticulata Blanco [ Rutaceae, Pericarpium Citri Reticulatae ], Saposhnikovia divaricata (Turcz.) Schischk. [ Apiaceae, Radix Saposhnikoviae divaricatae ], Magnolia biondii Pamp. [ Magnoliaceae, Flos Magnoliae Biondii ], Schisandra chinensis (Turcz.) Baill. [ Magnoliaceae, Schisandrae Chinensis Fructus ], Glycyrrhiza uralensis Fisch.. [Leguminosae, Glycyrrhizae Radix et Rhizoma ] | Liquiritin | 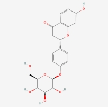 | Asthma-intraperitoneally injected[100 μg OVA + 1 mg AL(OH)3+ 0.2 mL NS)] and nebulized inhalation 2.5%OVA + nasally administered 105 TCID50 RSV | Firmicutes / Bacteroidetes (F/B)↑, Lachnospiraceae↑; Rikenellaaceae↓, Bifidobacteriaceae↓, Verrucomirobiaceae↓, Peptoccacea↓, Anaeroplastaceae↓, Unclassified Firmicutes↓, Alistipes↓ | Fecal SCFAs:acetic acid↑, propionic acid↑ | airway hyperreactivity↓, BALF inflammatory cell↓, Treg cell↑ | Gu-Ben-Fang-Xiao Decoction effectively alleviated the remission symptoms of asthma by regulating the gut microbiota-acetate-Tregs axis. | (Lu et al., 2016; Dong et al., 2020) |
|  |  |  |  |  |  |  |  |  |  |  |
|  |  |  |  |  |  |  |  |  |  |  |
|  |  |  |  |  |  |  |  |  |  |  |
|  |  |  | Prim-O-glucosylcimifugin | 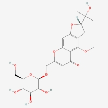 |  |  |  |  |  |  |
|  |  |  |  |  |  |  |  |  |  |  |
|  |  |  |  |  |  |  |  |  |  |  |
|  |  |  |  |  |  |  |  |  |  |  |
|  |  |  | Hesperidin | 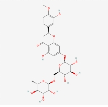 |  |  |  |  |  |  |
|  |  |  |  |  |  |  |  |  |  |  |
|  |  |  |  |  |  |  |  |  |  |  |
|  |  |  |  |  |  |  |  |  |  |  |
|  |  |  |  |  |  |  |  |  |  |  |
|  |  |  | Lobetyolin | 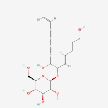 |  |  |  |  |  |  |
|  |  |  |  |  |  |  |  |  |  |  |
|  |  |  |  |  |  |  |  |  |  |  |
|  |  |  |  |  |  |  |  |  |  |  |
|  |  |  | Magnolin | 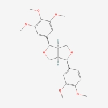 |  |  |  |  |  |  |
|  |  |  |  |  |  |  |  |  |  |  |
|  |  |  |  |  |  |  |  |  |  |  |
|  |  |  |  |  |  |  |  |  |  |  |
|  |  |  | Schizandrin | 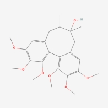 |  |  |  |  |  |  |
|  |  |  |  |  |  |  |  |  |  |  |
|  |  |  |  |  |  |  |  |  |  |  |
|  |  |  |  |  |  |  |  |  |  |  |
|  | Pentaherbs formula | *Lonicera japonica* Thunb. [Caprifoliaceae, Flos Lonicerae ], Mentha canadensis Linnaeus [Labiatae, herba Menthae ], Paeonia suffruticosa Andr. [ Ranunculaceae, Cortex Moutan ], Atractylodes lancea( Thunb.)DC. [Compositae, Rhizoma Atractylodis ], Phellodendron chinense Schneid. [ Rutaceae, Phellodendron amurense ] | Gallic Acid | 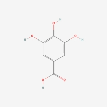 | Allergic Asthma-intraperitoneally injected 1% OVA in Imject Alum Adjuvant and nebulized inhalation 10mg/mL OVA | Firmicutes / Bacteroidetes (F/B)↓, Saccharibacteria↑, Butyricicoccus↓, Eubacterium_nodatum ↓, Lachnospiraceae UCG-006↓, Saccharibacteria_Saccharimonas↑, | SCFAs:isobutyric acid ↓ acetic acid↑, propionic acid↑, butyric acid↑ | airway hyperreactivity↓, IgE↓, airway remodeling↓; Total number of BALF eosinophils and neutrophils↓, IL-4↓, IL-31↓, IL-5↓, CCL5↓, IL-33↓, TGF-β↓, IL-13↓; spleen:CD4+CD25highFoxp3+Treg cell↑; blood serum:IL-10↑ | Pentaherbs formula alleviated allergic asthma symptoms in mice by inhibiting immune cells, reducing airway hyperresponsiveness and remodeling, and curbing goblet cell proliferation. It also adjusted spleen regulatory T cells, boosted serum IL-10 levels, and altered intestinal microbiota and short-chain fatty acids. | (Tsang et al., 2016, 2018) |
|  |  |  |  |  |  |  |  |  |  |  |
|  |  |  |  |  |  |  |  |  |  |  |
|  |  |  |  |  |  |  |  |  |  |  |
|  |  |  | Chlorogenic Acid | 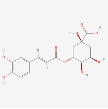 |  |  |  |  |  |  |
|  |  |  |  |  |  |  |  |  |  |  |
|  |  |  |  |  |  |  |  |  |  |  |
|  |  |  |  |  |  |  |  |  |  |  |
|  |  |  |  |  |  |  |  |  |  |  |
|  |  |  | Berberine | 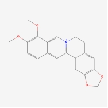 |  |  |  |  |  |  |
|  |  |  |  |  |  |  |  |  |  |  |
|  |  |  |  |  |  |  |  |  |  |  |
|  |  |  |  |  |  |  |  |  |  |  |
|  | Ephedra sinica polysaccharide | Ephedraceae, Herba Ephedrae | D-Glucose | 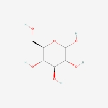 | Asthma-like Respiratory Disease-intraperitoneally injected [OVA and AL(OH)3] and nebulized inhalation 1% OVA＋PM2.5 | Firmicutes / Bacteroidetes↑, Proteobacteria↑, Lactic acid bacillus ↑, Prevotella↑, Butyl acid cocci↑, Paraprevotella↑, Enterococcus ↓, Ruminococcus ↓; Amaricoccus↑, Parabacteroides↑, Actinomyces ↑, Rhizobium ↑, Coxiellaceae↑ | SCFA:acetic acid↑, propionic acid↑, butyric acid↑, isobutyric acid ↑, pentanoic acid↑, isopentoic acid↑, isocaproic acid ↑ | BALF inflammatory cell↓, TNF-α↓, IgE↓, IL-1β↓, IL-6↓, Lung tissue injury↓, lung: NF-κB↓, TNF-α↓, IL-1β↓ | Ephedra sinica polysaccharide polysaccharide can alleviate allergic asthma symptoms in mice by reducing eosinophils in BALF, lowering serum Ig-E, IL-6, TNF-α, and IL-1β levels, inhibiting the NF-κB pathway, and balancing intestinal flora and SCFA. Thus, it shows potential for preventing and treating PM2.5 and ovalbumin-induced asthma-like diseases. | (Soua et al., 2020; Jx et al., 2022) |
|  |  |  |  |  |  |  |  |  |  |  |
|  |  |  |  |  |  |  |  |  |  |  |
|  |  |  |  |  |  |  |  |  |  |  |
|  |  |  | (2R,3S,4S,5R)-2,3,4,5,6-pentahydroxyhexanal | 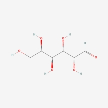 |  |  |  |  |  |  |
|  |  |  |  |  |  |  |  |  |  |  |
|  |  |  |  |  |  |  |  |  |  |  |
|  |  |  |  |  |  |  |  |  |  |  |
|  |  |  |  |  |  |  |  |  |  |  |
|  |  |  | D-Mannose | 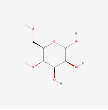 |  |  |  |  |  |  |
|  |  |  |  |  |  |  |  |  |  |  |
|  |  |  |  |  |  |  |  |  |  |  |
|  |  |  |  |  |  |  |  |  |  |  |
|  |  |  |  |  |  |  |  |  |  |  |
|  |  |  | L-Arabinose | 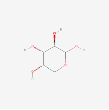 |  |  |  |  |  |  |
|  |  |  |  |  |  |  |  |  |  |  |
|  |  |  |  |  |  |  |  |  |  |  |
|  |  |  |  |  |  |  |  |  |  |  |
|  |  |  |  |  |  |  |  |  |  |  |
|  |  |  | Gluconic Acid | 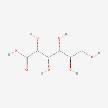 |  |  |  |  |  |  |
|  |  |  |  |  |  |  |  |  |  |  |
|  |  |  |  |  |  |  |  |  |  |  |
|  |  |  |  |  |  |  |  |  |  |  |
|  |  |  | D-Galacturonic Acid | 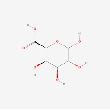 |  |  |  |  |  |  |
|  |  |  |  |  |  |  |  |  |  |  |
|  |  |  |  |  |  |  |  |  |  |  |
|  |  |  |  |  |  |  |  |  |  |  |
|  |  |  |  |  |  |  |  |  |  |  |
|  | Inulin | - | Inulin | 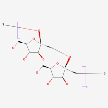 | Asthma-intraperitoneally injected [1mg OVA and 200mg AL(OH)3] and nebulized inhalation 1% OVA | parent substance :Bifidobacteriaceae↑, Lctobacteriaceae ↑, Dantoxicaceae↑, Lactobacillus↑, Blautia↑, Prevotella↑;Offspring :Lactobacteriaceae ↑, Enterobacteriaceae ↑, shiga's bacillus↑,Lactobacillus↓, Bacillus bifidus ↓ | Maternal SCFAs:acetic acid↑ , propionic acid↑, isobutyric acid↓, butanoic acid↓, Isovaleric acid↓, valeric acid↓, isohexanoic acid↑, hexanoic acid↓ | Offspring:IL-4↓; IL17↓; IgE↓; INF-γ↑ ; GPR41↑; GPR43↑; Degree of inflammatory cell infiltration in the bronchial and perivascular regions↓, Effluent material in the pulmonary interstitial and alveolar spaces↓ | Maternal inulin intake during pregnancy alters the gut microbiome, boosting SCFA-producing bifidobacteria and reducing offspring's asthma-related inflammation. It also regulates the offspring's gut microbiota before asthma onset, indicating its crucial role in asthma development. | (Qin et al., 2023; Yuan et al., 2023) |
|  |  |  |  |  |  |  |  |  |  |  |
|  |  |  |  |  |  |  |  |  |  |  |
|  |  |  |  |  |  |  |  |  |  |  |
|  |  |  |  |  |  |  |  |  |  |  |
| Amino acid | Right return pill drug | *Rehmannia glutinosa* Libosch. [Scrophulariaceae, Rehmanniae Radix ], Dioscorea opposita Thunb. [Dioscoreaceae, Dioscoreae Rhizoma ], Cornus officinalis Sieb. et Zucc. [Cornaceae, Corni Fructus ], Lycium barbarum L. [Solanaceae, Lycii Fructus ], Cuscuta chinensis Lam. [Convolvulaceae, Semen Cuscutae ], Cervus nippon Temminck [Cervidae , Cervi Cornu Pantotrichum ], Eucommia ulmoides Oliv. [Eucommiaceae, Eucommiae Cortex ], Cinnamomum cassia Presl [Lauraceae, Cinnamomi Cortex ], Angelica sinensis (Oliv.) Diels [Umbelliferae, Angelicae Sinensis Radix ], Aconitum carmichaeli Debx. [Ranunculaceae, Aconiti Lateralis Radix Praeparata ] | Gallic acid | 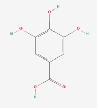 | Allergic Asthma-intratracheally administered Dust mite extract (1 mg/mL, 50 μL) 5 dose | Firmicutes / Bacteroidetes(F/B)↓, Eubacterium ↑, Blautia genus↑, Ruminobacter↑, Leptospirillum_NK4A136_group↑, Lactobacillus↓, Ruminococcaceae↓, Alistipes↑, Akkermansia↓, Blautia↑, Ruminiclostridium↑, Bacteroides↓, Candidatus↓, Streptococcus↓, Staphylococcus↓, Desulfovibrio↓, Bifidobacterium↑, Fusobacterium↓ | acetylcarnitine↑;carnitine↑;hypoxanthine↑;tryptophan↑;phenylalanine↑;norleucine↑;isoleucine↑;betaine;methionine↑;valine↑ | airway hyperresponsiveness↓, blood serum : IgE↓, TNF-α↓ | Right return pill drug plays a role in the treatment of allergic asthma by regulating amino acid metabolism disorders and improving intestinal flora imbalance. | (Wang et al., 2015; Hsu et al., 2021) |
|  |  |  |  |  |  |  |  |  |  |  |
|  |  |  |  |  |  |  |  |  |  |  |
|  |  |  |  |  |  |  |  |  |  |  |
|  |  |  | 5-Hydroxymethylfurfural | 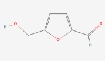 |  |  |  |  |  |  |
|  |  |  |  |  |  |  |  |  |  |  |
|  |  |  | Morroniside | 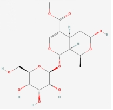 |  |  |  |  |  |  |
|  |  |  |  |  |  |  |  |  |  |  |
|  |  |  |  |  |  |  |  |  |  |  |
|  |  |  |  |  |  |  |  |  |  |  |
|  |  |  | Sweroside | 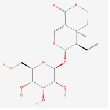 |  |  |  |  |  |  |
|  |  |  |  |  |  |  |  |  |  |  |
|  |  |  |  |  |  |  |  |  |  |  |
|  |  |  |  |  |  |  |  |  |  |  |
|  |  |  | Loganin | 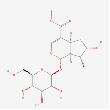 |  |  |  |  |  |  |
|  |  |  |  |  |  |  |  |  |  |  |
|  |  |  |  |  |  |  |  |  |  |  |
|  |  |  |  |  |  |  |  |  |  |  |
|  |  |  | Rutin | 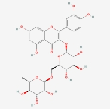 |  |  |  |  |  |  |
|  |  |  |  |  |  |  |  |  |  |  |
|  |  |  |  |  |  |  |  |  |  |  |
|  |  |  |  |  |  |  |  |  |  |  |
|  |  |  | Cinnamic acid | 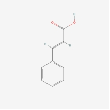 |  |  |  |  |  |  |
|  |  |  |  |  |  |  |  |  |  |  |
|  |  |  |  |  |  |  |  |  |  |  |
|  |  |  |  |  |  |  |  |  |  |  |
|  |  |  |  |  |  |  |  |  |  |  |
|  |  |  | Quercetin | 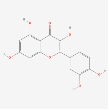 |  |  |  |  |  |  |
|  |  |  |  |  |  |  |  |  |  |  |
|  |  |  |  |  |  |  |  |  |  |  |
|  |  |  |  |  |  |  |  |  |  |  |
|  |  |  |  |  |  |  |  |  |  |  |
|  |  |  | Kaempferide | 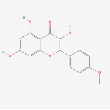 |  |  |  |  |  |  |
|  |  |  |  |  |  |  |  |  |  |  |
|  |  |  |  |  |  |  |  |  |  |  |
|  |  |  |  |  |  |  |  |  |  |  |
|  | Qing-Fei-Shen-Shi Decoction | *Prunus armeniaca* L. var. ansu Maxim. [Rosaceae, Armeniacae Semen Amarum ], Gypsum [Sulfates, Gypsum Fibrosum ], Smilax glabra Roxb. [ Liliaceae, Smilacis Glabrae Rhizoma ], Coix lacryma-jobi L. var. mayuen (Roman.)Stapf [Gramineae, Coicis Semen ], Benincasa hispida (Thunb.) Cogn. [Cucurbitaceae, Semen Benincasae ], Plantago asiatica L. [Plantaginaceae, Plantaginis Herba ], Pyrrosia sheareri (Bak.) Ching [Polypodiaceae, Pyrrosiae Folium ], Houttuynia cordata Thunb. [Saururaceae, Houttuyniae Herba ], Fritillaria thunbergii Miq. [Liliaceae, Fritillariae Thunbergii Bulbus ], Cryptotympana pustulata Fabricius [Cicadidae, Cicadae Periostracum ], and Glycyrrhiza uralensis Fisch.. [Leguminosae, Glycyrrhizae Radix et Rhizoma ] | - | - | Asthma-intraperitoneally injected(40 μg OVA + 1 mg of 10% alum + 200 μL of saline) and nebulized inhalation 2%OVA | *Firmicutes* / Bacteroidetes(F/B)↓, Bacterium lacticum ↑, Dubosie bacteria↑,Leptospirillum_NK4A136_group↓, helicobacter pylori↓ | 4-guanidinobutyric acid↓;L-ornithine↑;proline↑;2-deoxyuridine↓;uracil↓;uridine↓ | airway hyperreactivity↓, Wet-dry weight ratio of lung tissue(W/D)↓, BALF total protein content↓, Total cell counts of BALF↓, eosinocyte↓; blood serum: IgE↓, Damage of the bronchial tissue↓, IL-4↓, IL-5↓, IL-13↓, IFN-γ↑, SOD↑, GSH-Px↑ | Qing-Fei-Shen-Shi Decoction in improving asthma may be related to the regulation of intestinal microflora and arginine and proline metabolism and pyrimidine metabolism. | (H et al., 2023) |
|  |  |  |  |  |  |  |  |  |  |  |
|  | Guominkang formula | *Prunus mume*(Sieb.)Sieb.et Zuce. [Rosaceae, Fructus Mume ], Cryptotympana pustulata Fabricius [Cicadidae, Cicadae Periostracum ], Saposhnikovia divaricata (Turcz.) Schischk. [ Apiaceae, Radix Saposhnikoviae divaricatae ], Ganoderma lucidum (Leyss.ex Fr.) Karst. [Polyporaceae, Ganoderma ], Polygonum multiflorum Thunb. [Polygonaceae, Polygoni Multiflori Radix ], Gastrodia elata Bl. [ Orchidaceae, Gastrodiae Rhizoma ] | Cimicifugoside | 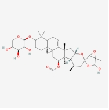 | Allergic Asthma-intraperitoneally injected (2 mg OVA and 2 mg Alum Adjuvant) and nebulized inhalation 1% OVA | Muriculum↑, Clostridium↑, klebsiella↑, Sdesulfovibrio↑, Rikenellaceae_RC9_gut_group↑, Chitinophageaceae↑, Cardioidaceae↑, Corynebacterium↑ | DL-glutamine↑;L-pyroglutamic acid↑;prostaglandin b1↓;prostaglandin e2↓;3,4-dihydroxyhydrocinnamic acid↓ | airway hyperreactivity↓; Inflammatory cell infiltration in the lung tissue↓, eosinophile granulocyte↓; IgE↓, IL-5↓, IL-13↓; IL-4↓, IFN-γ↑; IL-17A↓, IL-6↓, IL-10↑, TGF-β↑, Foxp3↑, Treg↑; RORγt ↓, Th17↓ | Guominkang formula modulates the recruitment of inflammatory cells and diminishes mucus production and airway resistance by regulating the immune balance between Th1/Th2 and Treg/Th17 cells. Furthermore, GMK induces alterations in the gut microbiota of allergic asthma mouse models, with specific metabolic biomarkers identified as being associated with the balance of immune cells. | (Tang et al., 2022; Zhou et al., 2022a) |
|  |  |  |  |  |  |  |  |  |  |  |
|  |  |  |  |  |  |  |  |  |  |  |
|  |  |  |  |  |  |  |  |  |  |  |
|  |  |  | 2,3,5,4'-tetrahydroxystilbene-2-O-beta-D-glucoside | 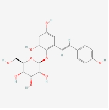 |  |  |  |  |  |  |
|  |  |  |  |  |  |  |  |  |  |  |
|  |  |  |  |  |  |  |  |  |  |  |
|  |  |  |  |  |  |  |  |  |  |  |
|  |  |  | Cimifugin | 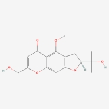 |  |  |  |  |  |  |
|  |  |  |  |  |  |  |  |  |  |  |
|  |  |  |  |  |  |  |  |  |  |  |
|  |  |  |  |  |  |  |  |  |  |  |
|  |  |  | 5-O-Methylvisammioside | 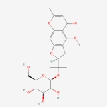 |  |  |  |  |  |  |
|  |  |  |  |  |  |  |  |  |  |  |
|  |  |  |  |  |  |  |  |  |  |  |
|  |  |  | Emodin | 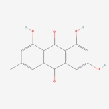 |  |  |  |  |  |  |
|  |  |  |  |  |  |  |  |  |  |  |
|  |  |  |  |  |  |  |  |  |  |  |
|  |  |  | Pentosalen | 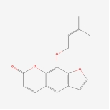 |  |  |  |  |  |  |
|  |  |  |  |  |  |  |  |  |  |  |
|  |  |  |  |  |  |  |  |  |  |  |
|  |  |  |  |  |  |  |  |  |  |  |
|  |  |  | Emodin | 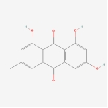 |  |  |  |  |  |  |
|  |  |  |  |  |  |  |  |  |  |  |
|  |  |  |  |  |  |  |  |  |  |  |
|  |  |  | Kaempferol | 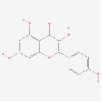 |  |  |  |  |  |  |
|  |  |  |  |  |  |  |  |  |  |  |
|  |  |  |  |  |  |  |  |  |  |  |
|  |  |  | Ursolic Acid | 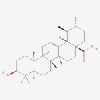 |  |  |  |  |  |  |
|  |  |  |  |  |  |  |  |  |  |  |
|  |  |  |  |  |  |  |  |  |  |  |
|  |  |  | Citric Acid | 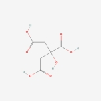 |  |  |  |  |  |  |
|  |  |  |  |  |  |  |  |  |  |  |
|  |  |  |  |  |  |  |  |  |  |  |
|  |  |  | Malic Acid | 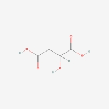 |  |  |  |  |  |  |
|  |  |  |  |  |  |  |  |  |  |  |
|  |  |  |  |  |  |  |  |  |  |  |
|  |  |  |  |  |  |  |  |  |  |  |
|  |  |  | Gallic Acid | 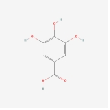 |  |  |  |  |  |  |
|  |  |  |  |  |  |  |  |  |  |  |
|  |  |  |  |  |  |  |  |  |  |  |
|  |  |  |  |  |  |  |  |  |  |  |
|  |  |  | Hamaudol | 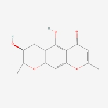 |  |  |  |  |  |  |
|  |  |  |  |  |  |  |  |  |  |  |
|  |  |  |  |  |  |  |  |  |  |  |
|  |  |  |  |  |  |  |  |  |  |  |
|  |  |  | Resveratrol | 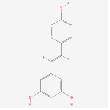 |  |  |  |  |  |  |
|  |  |  |  |  |  |  |  |  |  |  |
|  |  |  |  |  |  |  |  |  |  |  |
|  |  |  |  |  |  |  |  |  |  |  |
|  | Tuo-Min-Ding-Chuan decoction | *Ephedra sinica* Stapf. [ Ephedraceae, Herba Ephedrae ], Prunus armeniaca L. var. ansu Maxim. [Rosaceae, Armeniacae Semen Amarum ], Gypsum [Sulfates, Gypsum Fibrosum ], Glycyrrhiza uralensis Fisch. [ Leguminosae , Glycyrrhizae Radix et Rhizoma ], Saposhnikovia divaricata (Turcz.) Schischk. [ Apiaceae, Radix Saposhnikoviae divaricatae ], Ganoderma lucidum (Leyss.ex Fr.) Karst. [Polyporaceae, Ganoderma ], Prunus mume(Sieb.)Sieb.et Zuce. [Rosaceae, Fructus Mume ], Cryptotympana pustulata Fabricius [Cicadidae, Cicadae Periostracum ], Fagopyrum dibotrys (D. Don) Hara [Polygonaceae, Fagopyri Dibotryis Rhizoma ], Polygonum multiflorum Thunb. [Polygonaceae, Polygoni Multiflori Radix ], Gastrodia elata Bl. [ Orchidaceae, Gastrodiae Rhizoma ], Bombyx mori Linnaeus [ Silkworm pilgrimaging, Bombyx Batryticatus ] | Imidazoleacetic acid | 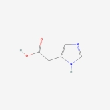 | Eosinophilic Asthma-intraperitoneally injected (2 mg of OVA and 2 mg Alum Adjuvant ) and nebulized inhalation 1% OVA | Rikenellaceae_RC9_gut_group↑, Bifidobacterium↑, Rikenella↑, Butyricimonas↑, Prevotella↑, Enterococcus↑, Peptoniphilus↑, Dialiste↑, Corynebacterium↑, Dermabacter↑, Varibaculum ↑ | Imidazoleacetic acid↑; DL-glutamine↑; L-pyroglutamic acid↑; 2-deoxy-D-glucose↑ | eosinophile granulocyte ↓; airway hyperreactivity; airway resistance↓, IgE↓, BALF:IL-5↓, IL-4↓, IL-13↓; TGFβ↑和IL10↑; IL-6↓和IL17A↓, RORγt↓; spleen:Treg cell↑, Th17 cell↓ | Tuo-Min-Ding-Chuan decoction boosted Treg cells and reduced Th17 cells, easing asthma symptoms. The study also identified potential biomarkers among metabolites and gut microorganisms linked to Treg and Th17 cells, supporting the use of traditional Chinese medicine for treating allergic asthma. | (Qin et al., 2021; Zhou et al., 2022b) |
|  |  |  |  |  |  |  |  |  |  |  |
|  |  |  |  |  |  |  |  |  |  |  |
|  |  |  | DL-Glutamine | 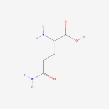 |  |  |  |  |  |  |
|  |  |  |  |  |  |  |  |  |  |  |
|  |  |  |  |  |  |  |  |  |  |  |
|  |  |  |  |  |  |  |  |  |  |  |
|  |  |  | 2-deoxy-D-glucose | 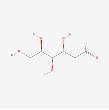 |  |  |  |  |  |  |
|  |  |  |  |  |  |  |  |  |  |  |
|  |  |  |  |  |  |  |  |  |  |  |
|  |  |  |  |  |  |  |  |  |  |  |
|  |  |  |  |  |  |  |  |  |  |  |
|  |  |  | L-Pyroglutamic acid | 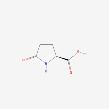 |  |  |  |  |  |  |
|  |  |  |  |  |  |  |  |  |  |  |
|  |  |  |  |  |  |  |  |  |  |  |
|  |  |  |  |  |  |  |  |  |  |  |
|  |  |  |  |  |  |  |  |  |  |  |
| Pyrimidine | Pingchuan formula | *Ephedra sinica* Stapf. [ Ephedraceae, Herba Ephedrae ], Glycyrrhiza uralensis Fisch. [ Leguminosae , Glycyrrhizae Radix et Rhizoma ], Sinapis alba L. [ Brassicaceae, Sinapis alba ], Prunus armeniaca L. var. ansu Maxim. [Rosaceae, Armeniacae Semen Amarum ], Perilla frutescens (L.) Britt. [ Labiatae, Perilla Frutescens ], Scutellaria baicalensis Georgi [ Lamiaceae , Scutellariae Radix ], Prunus persica (L.) Batsch [ Rosaceae, Persicae Semen ], Pheretima aspergillum (E. Perrier) [ Megascolecidae, Pheretima] | Hydroquinone | 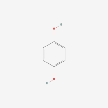 | Asthma-intraperitoneally injected [0.8mg/mL OVA and 100μL AL(OH)3] and nebulized inhalation 5% OVA | Clostridia_UCG_014↑, Verrucomicrobiales↑, Akkermansiaceae↑, Verrucomicrobiae↑, Verrucomicrobiota↑, Akkermansia↑, Clostridium_IV↑, Clostridium_XIVa↑, Akkmansia↑, Roseburia↑; Blautia↓, Barnesiella↓, Clostridium_III↓ | acacetin↑; abscisic acid↑; pyrimidine↓; phenylpropanoids↓; pantothenate↓; CoA biosynthesis↓; bataalanine↓ | IL-18↑, IL-4↓, IL-6↓, Eotaxin↓ | The aqueous extract of Pingchuan Decoction has been shown to reduce the levels of inflammatory mediators, including IL-18, IL-6, IL-4, and eosinophil chemokines, in lung tissue. Additionally, it modulates serum metabolites in mice, influences the pyrimidine metabolic pathway, and alters the composition of the intestinal microbiota. These findings indicate that Pingchuan Decoction may alleviate asthma symptoms in mice through the regulation of serum metabolites and intestinal microbiota, thereby offering a novel perspective on its therapeutic potential. | (Liu et al., 2023a) |
|  |  |  |  |  |  |  |  |  |  |  |
|  |  |  |  |  |  |  |  |  |  |  |
|  |  |  |  |  |  |  |  |  |  |  |
|  |  |  |  |  |  |  |  |  |  |  |
|  |  |  | Kaempferol | 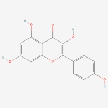 |  |  |  |  |  |  |
|  |  |  |  |  |  |  |  |  |  |  |
|  |  |  |  |  |  |  |  |  |  |  |
|  |  |  |  |  |  |  |  |  |  |  |
|  |  |  | Catechol | 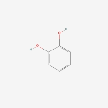 |  |  |  |  |  |  |
|  |  |  |  |  |  |  |  |  |  |  |
|  |  |  |  |  |  |  |  |  |  |  |
|  |  |  |  |  |  |  |  |  |  |  |
|  |  |  |  |  |  |  |  |  |  |  |
|  |  |  | Acetoacetic acid | 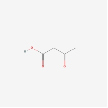 |  |  |  |  |  |  |
|  |  |  |  |  |  |  |  |  |  |  |
|  |  |  |  |  |  |  |  |  |  |  |
|  |  |  |  |  |  |  |  |  |  |  |
|  |  |  |  |  |  |  |  |  |  |  |
|  |  |  | Oleic Acid | 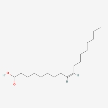 |  |  |  |  |  |  |
|  |  |  |  |  |  |  |  |  |  |  |
|  |  |  |  |  |  |  |  |  |  |  |
|  |  |  |  |  |  |  |  |  |  |  |
|  |  |  |  |  |  |  |  |  |  |  |
|  |  |  | Ephedrine | 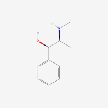 |  |  |  |  |  |  |
|  |  |  |  |  |  |  |  |  |  |  |
|  |  |  |  |  |  |  |  |  |  |  |
|  |  |  |  |  |  |  |  |  |  |  |
|  |  |  | Choline | 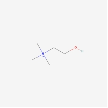 |  |  |  |  |  |  |
|  |  |  |  |  |  |  |  |  |  |  |
|  |  |  |  |  |  |  |  |  |  |  |
|  |  |  |  |  |  |  |  |  |  |  |
|  |  |  |  |  |  |  |  |  |  |  |
|  |  |  | 4-Methyl-2-oxopentanoic acid | 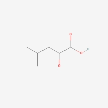 |  |  |  |  |  |  |
|  |  |  |  |  |  |  |  |  |  |  |
|  |  |  |  |  |  |  |  |  |  |  |
|  |  |  |  |  |  |  |  |  |  |  |
|  |  |  |  |  |  |  |  |  |  |  |
|  |  |  | Aspartic Acid | 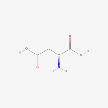 |  |  |  |  |  |  |
|  |  |  |  |  |  |  |  |  |  |  |
|  |  |  |  |  |  |  |  |  |  |  |
|  |  |  |  |  |  |  |  |  |  |  |
|  |  |  |  |  |  |  |  |  |  |  |
|  |  |  | Gamma-Aminobutyric Acid | 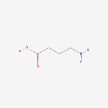 |  |  |  |  |  |  |
|  |  |  |  |  |  |  |  |  |  |  |
|  |  |  |  |  |  |  |  |  |  |  |
|  |  |  |  |  |  |  |  |  |  |  |
|  |  |  |  |  |  |  |  |  |  |  |
|  |  |  | Serine | 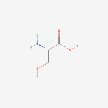 |  |  |  |  |  |  |
|  |  |  |  |  |  |  |  |  |  |  |
|  |  |  |  |  |  |  |  |  |  |  |
|  |  |  |  |  |  |  |  |  |  |  |
|  |  |  |  |  |  |  |  |  |  |  |
|  |  |  | Chrysosplenetin | 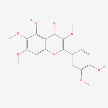 |  |  |  |  |  |  |
|  |  |  |  |  |  |  |  |  |  |  |
|  |  |  |  |  |  |  |  |  |  |  |
|  |  |  |  |  |  |  |  |  |  |  |
|  |  |  |  |  |  |  |  |  |  |  |
|  |  |  | 1,4-Diaminobutane | 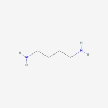 |  |  |  |  |  |  |
|  |  |  |  |  |  |  |  |  |  |  |
|  |  |  |  |  |  |  |  |  |  |  |
|  |  |  |  |  |  |  |  |  |  |  |
|  |  |  | Oxazepam | 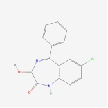 |  |  |  |  |  |  |
|  |  |  |  |  |  |  |  |  |  |  |
|  |  |  |  |  |  |  |  |  |  |  |
|  |  |  |  |  |  |  |  |  |  |  |
|  |  |  | Fomepizole | 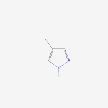 |  |  |  |  |  |  |
|  |  |  |  |  |  |  |  |  |  |  |
|  |  |  |  |  |  |  |  |  |  |  |
|  |  |  |  |  |  |  |  |  |  |  |
|  |  |  | Phenyl acetate | 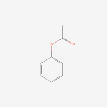 |  |  |  |  |  |  |
|  |  |  |  |  |  |  |  |  |  |  |
|  |  |  |  |  |  |  |  |  |  |  |
|  |  |  |  |  |  |  |  |  |  |  |
|  |  |  | Guanosine | 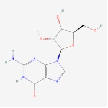 |  |  |  |  |  |  |
|  |  |  |  |  |  |  |  |  |  |  |
|  |  |  |  |  |  |  |  |  |  |  |
|  |  |  |  |  |  |  |  |  |  |  |
|  |  |  | Phenylephrine | 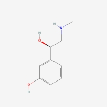 |  |  |  |  |  |  |
|  |  |  |  |  |  |  |  |  |  |  |
|  |  |  |  |  |  |  |  |  |  |  |
|  |  |  |  |  |  |  |  |  |  |  |
| Bile acid | Cordyceps militaris polysaccharide | Clavicipitaceae, Cordyceps | L-Rhamnose | 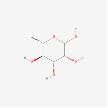 | Allergic Asthma-intraperitoneally injected [50μg OVA and 4mg AL(OH)3] and nebulized inhalation 1% OVA | Firmicutes / Bacteroidetes(F/B)↑; Lachnospiraceae ↑, Alistipes↑, Muribaculaceae↓, Akkermansia↑, s_unculture_bacteria_g_UCG-005 ↑, f_Anaerofustaceae↑, o_Eubacteriaes↑, s_unclassifed_g_Anaerofustis↑, g_UCG - 005↑, g_Anaerofustis↑ | bile acid↓; cholic acid↓; chenodeoxycholic acid↓; deoxycholic acid↑; lithocholic acid↑ | blood serum :IgE↓, TNF-α↓, IL-4↓, IL-5↓, IL-6↓, IL-13↓, IL17A↓, IFN-γ↑; The severity of the inflammatory cell infiltration↓; NF-κB↓, IκBα↓, IKKα↓, IKKβ↓; Abnormal tissue damage of the duodenum↓ | Cordyceps militaris polysaccharide mitigates oxidative stress and inflammatory responses in allergic asthma through the modulation of intestinal microbiota and its metabolites, including bile acids, vitamin B, and genes associated with lipopolysaccharide biosynthesis. | (Dong et al., 2015; Song et al., 2023) |
|  |  |  |  |  |  |  |  |  |  |  |
|  |  |  |  |  |  |  |  |  |  |  |
|  |  |  |  |  |  |  |  |  |  |  |
|  |  |  |  |  |  |  |  |  |  |  |
|  |  |  | L-Arabinose | 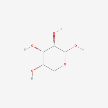 |  |  |  |  |  |  |
|  |  |  |  |  |  |  |  |  |  |  |
|  |  |  |  |  |  |  |  |  |  |  |
|  |  |  |  |  |  |  |  |  |  |  |
|  |  |  |  |  |  |  |  |  |  |  |
|  |  |  | 2-acetamido-2-deoxy-alpha-D-galactopyranose | 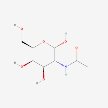 |  |  |  |  |  |  |
|  |  |  |  |  |  |  |  |  |  |  |
|  |  |  |  |  |  |  |  |  |  |  |
|  |  |  |  |  |  |  |  |  |  |  |
|  |  |  |  |  |  |  |  |  |  |  |
|  |  |  | D-Galactose | 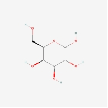 |  |  |  |  |  |  |
|  |  |  |  |  |  |  |  |  |  |  |
|  |  |  |  |  |  |  |  |  |  |  |
|  |  |  |  |  |  |  |  |  |  |  |
|  |  |  | D-Glucose | 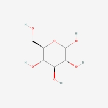 |  |  |  |  |  |  |
|  |  |  |  |  |  |  |  |  |  |  |
|  |  |  |  |  |  |  |  |  |  |  |
|  |  |  |  |  |  |  |  |  |  |  |
|  |  |  |  |  |  |  |  |  |  |  |
|  |  |  | D-Xylose | 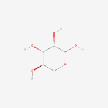 |  |  |  |  |  |  |
|  |  |  |  |  |  |  |  |  |  |  |
|  |  |  |  |  |  |  |  |  |  |  |
|  |  |  |  |  |  |  |  |  |  |  |
|  |  |  | D-Glucuronic Acid | 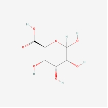 |  |  |  |  |  |  |
|  |  |  |  |  |  |  |  |  |  |  |
|  |  |  |  |  |  |  |  |  |  |  |
|  |  |  |  |  |  |  |  |  |  |  |
|  |  |  |  |  |  |  |  |  |  |  |
| Unmeasured | Dachengqi Decoction | *Rheum palatum* L. [ Polygonaceae, Radix et Rhizoma Rhei ], Magnolia officinalis Rehd.et Wils. [ Magnoliaceae, Magnolia officinalis ], Mirabilite [ Sulphate, Natrii Sulfas ] | Quercetin | 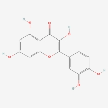 | Bronchial Asthma-intraperitoneally injected (0.2 mL containing 2 mg aluminum hydroxide and 20 mg OVA) and nebulized inhalation 1% OVA | Small intestine and colon: Allobaculum↑, Romboutsia↑, Turicibacter↑; jejunum and ileum:Faecalibaculum↓, Clostridium_sensu_stricto_1↑; jejunum :Senegalimassilia↑, Lactobacillus_vaginalis ↓; colon:Lactobacillus_gasseri↑ | - | BALF:IgE↓, IL-4↓, IL-5↓, Water content of feces↑, Colon length↑; Intestinal epithelial injury↓, inflammatory infiltration ↓; Small intestine, colon:ILC-2↓ | Dachengqi Decoction mitigates the accumulation of ILC2 cells across various intestinal regions in a microbiota-dependent manner, consequently alleviating concurrent intestinal inflammation associated with OVA-induced asthma. | (Yin et al., 2022; Liu et al., 2023b) |
|  |  |  |  |  |  |  |  |  |  |  |
|  |  |  |  |  |  |  |  |  |  |  |
|  |  |  |  |  |  |  |  |  |  |  |
|  |  |  | Emodin | 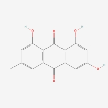 |  |  |  |  |  |  |
|  |  |  |  |  |  |  |  |  |  |  |
|  |  |  |  |  |  |  |  |  |  |  |
|  |  |  |  |  |  |  |  |  |  |  |
|  |  |  |  |  |  |  |  |  |  |  |
|  |  |  | Luteolin | 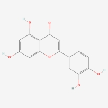 |  |  |  |  |  |  |
|  |  |  |  |  |  |  |  |  |  |  |
|  |  |  |  |  |  |  |  |  |  |  |
|  |  |  |  |  |  |  |  |  |  |  |
|  |  |  | Rhein | 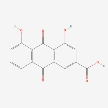 |  |  |  |  |  |  |
|  |  |  |  |  |  |  |  |  |  |  |
|  |  |  |  |  |  |  |  |  |  |  |
|  |  |  |  |  |  |  |  |  |  |  |
|  |  |  |  |  |  |  |  |  |  |  |
|  |  |  | Apigenin | 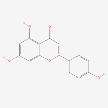 |  |  |  |  |  |  |
|  |  |  |  |  |  |  |  |  |  |  |
|  |  |  |  |  |  |  |  |  |  |  |
|  |  |  |  |  |  |  |  |  |  |  |
|  |  |  |  |  |  |  |  |  |  |  |
|  |  |  | Aloe emodin | 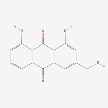 |  |  |  |  |  |  |
|  |  |  |  |  |  |  |  |  |  |  |
|  |  |  |  |  |  |  |  |  |  |  |
|  |  |  |  |  |  |  |  |  |  |  |
|  |  |  |  |  |  |  |  |  |  |  |
|  |  |  | Hesperidin | 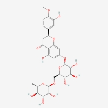 |  |  |  |  |  |  |
|  |  |  |  |  |  |  |  |  |  |  |
|  |  |  |  |  |  |  |  |  |  |  |
|  |  |  |  |  |  |  |  |  |  |  |
|  |  |  | Hesperetin | 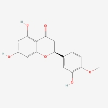 |  |  |  |  |  |  |
|  |  |  |  |  |  |  |  |  |  |  |
|  |  |  |  |  |  |  |  |  |  |  |
|  |  |  |  |  |  |  |  |  |  |  |
|  |  |  | Nobiletin | 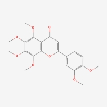 |  |  |  |  |  |  |
|  |  |  |  |  |  |  |  |  |  |  |
|  |  |  |  |  |  |  |  |  |  |  |
|  |  |  |  |  |  |  |  |  |  |  |
|  | Recuperating lung decoction | *Astragalus membranaceus* (Fisch.) Bunge [Leguminosae, Astragali Radix ], Cinnamomum cassia Presl [ Lauraceae, Cinnamomi Cortex ], Perilla frutescens (L.) Britt. [ Labiatae, Perilla Frutescens ], Schisandra chinensis (Turcz.) Baill. [ Magnoliaceae, Schisandrae Chinensis Fructus ], Zingiber officinale Rosc.[ Zingiberaceae , Zingiberis Rhizoma ], Cornus officinalis Sieb. et Zucc. [Cornaceae, Corni Fructus ], Prunus mume(Sieb.)Sieb.et Zuce. [Rosaceae, Fructus Mume ], Inula japonica Thunb. [ Asteraceae, Flos Inulae ], Magnolia officinalis Rehd.et Wils. [ Magnoliaceae, Magnolia officinalis ], Anemarrhena asphodeloides Bge. [ Liliaceae, Anemarrhenae Rhizoma ], Glycyrrhiza uralensis Fisch. [ Leguminosae , Glycyrrhizae Radix et Rhizoma ] | Valine | 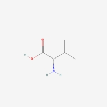 | Asthma-intraperitoneally injected [0.2 mL 10% OVA/AL(OH)3 and 0.0023% whooping cough toxoid] and nebulized inhalation 1% OVA | Prevotella↑, Adlercreutzia↑, Erysipelotrichaceae↓, Escherichia↑ | - | Symptoms of asthmatic behaviour↓, air way resistance↓; blood serum :IgE↓; BALF eosinophils↓, Inflammatory cells infiltrate in the lungs↓, Damage of the bronchial tissue↓, bronchial spasm↓, macrophage ↓; | Recuperating lung decoction regulates intestinal microbes in rats with allergic bronchial asthma. Oral RLD increases beneficial bacteria like lactic acid bacteria and bifidobacteria, making the microbial community in the RLD group similar to the control group, unlike the model group. This suggests Bufei Decoction may alleviate asthma symptoms by modulating gut microorganisms. | [(Kong et al., 2016)](#_ENREF_44) |
|  |  |  |  |  |  |  |  |  |  |  |
|  |  |  |  |  |  |  |  |  |  |  |
|  |  |  |  |  |  |  |  |  |  |  |
|  |  |  | Malic Acid | 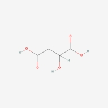 |  |  |  |  |  |  |
|  |  |  |  |  |  |  |  |  |  |  |
|  |  |  |  |  |  |  |  |  |  |  |
|  |  |  |  |  |  |  |  |  |  |  |
|  |  |  | Gluconic Acid | 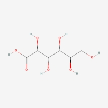 |  |  |  |  |  |  |
|  |  |  |  |  |  |  |  |  |  |  |
|  |  |  |  |  |  |  |  |  |  |  |
|  |  |  |  |  |  |  |  |  |  |  |
|  |  |  |  |  |  |  |  |  |  |  |
|  |  |  | D-Galactose | 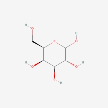 |  |  |  |  |  |  |
|  |  |  |  |  |  |  |  |  |  |  |
|  |  |  |  |  |  |  |  |  |  |  |
|  |  |  |  |  |  |  |  |  |  |  |
|  |  |  |  |  |  |  |  |  |  |  |
|  |  |  | Stearic Acid | 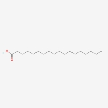 |  |  |  |  |  |  |
|  |  |  |  |  |  |  |  |  |  |  |
|  |  |  |  |  |  |  |  |  |  |  |
|  |  |  |  |  |  |  |  |  |  |  |
|  | Sijunzi Decoction | *Panax ginseng* C. A. Mey. [ Araliaceae, Ginseng Radix et Rhizoma ], Poria cocos (Schw.) Wolf [ Polyporaceae , Poria ], Atractylodes macrocephala Koidz.[ Asteraceae , Atractylodis Macrocephalae Rhizoma ], Glycyrrhiza uralensis Fisch. [ Leguminosae , Glycyrrhizae Radix et Rhizoma ] | Astilbin | 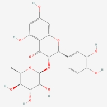 | Asthma-intraperitoneally injected (50 μg of OVA and 50 μg Imject Alum ) and nebulized inhalation 2% OVA | Firmicutes / Bacteroidetes(F/B)↑, norank_f__Muribaculacea↑, norank_f__Lachnospiraceae↑, Bacteroides↑, Monoglobus↑, Parabacteroides↑, Gemela↑, Lachnospiraceae_UCG-001↑ | - | Inflammatory cell infiltration in the lung tissue↓, Damage of the bronchial tissue↓; IFN-γ and IL-10↑, IL-4↓, IL-17↓, TLR2↓, TLR7↑ | Sijunzi Decoction has been demonstrated to mitigate airway inflammation and sustain intestinal homeostasis through the modulation of beneficial intestinal microbiota. Furthermore, the study elucidated the critical role of the TLR signaling pathway in mediating the effects of Sijunzi Decoction. | [(](#_ENREF_42)(Jia et al., 2023; Dai et al., 2024) |
|  |  |  |  |  |  |  |  |  |  |  |
|  |  |  |  |  |  |  |  |  |  |  |
|  |  |  |  |  |  |  |  |  |  |  |
|  |  |  | Liquiritin apioside | 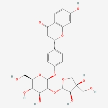 |  |  |  |  |  |  |
|  |  |  |  |  |  |  |  |  |  |  |
|  |  |  |  |  |  |  |  |  |  |  |
|  |  |  |  |  |  |  |  |  |  |  |
|  |  |  |  |  |  |  |  |  |  |  |
|  |  |  | Ononin | 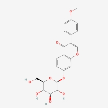 |  |  |  |  |  |  |
|  |  |  |  |  |  |  |  |  |  |  |
|  |  |  |  |  |  |  |  |  |  |  |
|  |  |  |  |  |  |  |  |  |  |  |
|  |  |  |  |  |  |  |  |  |  |  |
|  |  |  | Ginsenoside B2 | 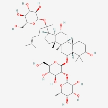 |  |  |  |  |  |  |
|  |  |  |  |  |  |  |  |  |  |  |
|  |  |  |  |  |  |  |  |  |  |  |
|  |  |  |  |  |  |  |  |  |  |  |
|  |  |  |  |  |  |  |  |  |  |  |
|  |  |  | Ginsenoside Rg1 | 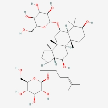 |  |  |  |  |  |  |
|  |  |  |  |  |  |  |  |  |  |  |
|  |  |  |  |  |  |  |  |  |  |  |
|  |  |  |  |  |  |  |  |  |  |  |
|  |  |  |  |  |  |  |  |  |  |  |
|  |  |  | Notoginsenoside R2 | 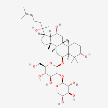 |  |  |  |  |  |  |
|  |  |  |  |  |  |  |  |  |  |  |
|  |  |  |  |  |  |  |  |  |  |  |
|  |  |  |  |  |  |  |  |  |  |  |
|  |  |  |  |  |  |  |  |  |  |  |
|  |  |  | Ginsenoside Rb1 | 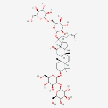 |  |  |  |  |  |  |
|  |  |  |  |  |  |  |  |  |  |  |
|  |  |  |  |  |  |  |  |  |  |  |
|  |  |  |  |  |  |  |  |  |  |  |
|  |  |  | Ginsenoside C | 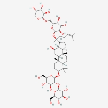 |  |  |  |  |  |  |
|  |  |  |  |  |  |  |  |  |  |  |
|  |  |  |  |  |  |  |  |  |  |  |
|  |  |  |  |  |  |  |  |  |  |  |
|  |  |  |  |  |  |  |  |  |  |  |
|  |  |  | Ginsenoside Ro | 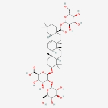 |  |  |  |  |  |  |
|  |  |  |  |  |  |  |  |  |  |  |
|  |  |  |  |  |  |  |  |  |  |  |
|  |  |  |  |  |  |  |  |  |  |  |
|  |  |  | Glycyrrhizin | 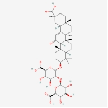 |  |  |  |  |  |  |
|  |  |  |  |  |  |  |  |  |  |  |
|  |  |  |  |  |  |  |  |  |  |  |
|  |  |  |  |  |  |  |  |  |  |  |
|  | Shaoyao-Gancao-Tang | *Paeonia lactiflora* Pall. [ Ranunculaceae, Paeoniae Radix Alba ], Glycyrrhiza uralensis Fisch. [ Leguminosae , Glycyrrhizae Radix et Rhizoma ] | Gallic Acid | 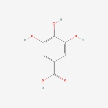 | Allergic Asthma-intraperitoneal and subcutaneous injection 1% OVA and 2% AL(OH)3 and nebulized inhalation 2% OVA | Firmicutes↑, Clostridia↑, GCA_900066575↑, Lachnospiraceae_NC2004_group↑, Oscillibacter↑, Ruminiclostridium_9↑, Ruminococcaceae_UCG_005↑, o_Desulfovibrionales↑, c_Deltaproteobacteria↑, Ruminococcus_UCG-005↓, Ruminococcus_2↓, Candidatus_Sacchrimonas↑ | - | BALF and blood serum:IgE↓, Inflammatory cell infiltration in the lung tissue↓, Damage of the bronchial tissue↓, Inflammatory cell infiltration in the colon↓; Lung, colon:IFN-γ↑, IL-4↓ | Shaoyao-Gancao-Tang has been shown to decrease the levels of IL-4, a cytokine secreted by Th2 cells, in both the lungs and intestines, while concurrently increasing the levels of IFN-γ, a cytokine secreted by Th1 cells. This modulation restores the IFN-γ/IL-4 ratio, thereby ameliorating asthma symptoms. Furthermore, Shaoyao-Gancao-Tang contributes to asthma improvement by modulating the gut microbiota, specifically by enhancing the abundance of microorganisms that exert beneficial effects on asthma management. | (He et al., 2023) |
|  |  |  |  |  |  |  |  |  |  |  |
|  |  |  |  |  |  |  |  |  |  |  |
|  |  |  |  |  |  |  |  |  |  |  |
|  |  |  |  |  |  |  |  |  |  |  |
|  |  |  | Albiflorin | 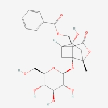 |  |  |  |  |  |  |
|  |  |  |  |  |  |  |  |  |  |  |
|  |  |  |  |  |  |  |  |  |  |  |
|  |  |  |  |  |  |  |  |  |  |  |
|  |  |  | Paeoniflorin | 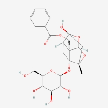 |  |  |  |  |  |  |
|  |  |  |  |  |  |  |  |  |  |  |
|  |  |  |  |  |  |  |  |  |  |  |
|  |  |  |  |  |  |  |  |  |  |  |
|  |  |  |  |  |  |  |  |  |  |  |
|  |  |  | Liquiritin apioside | 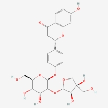 |  |  |  |  |  |  |
|  |  |  |  |  |  |  |  |  |  |  |
|  |  |  |  |  |  |  |  |  |  |  |
|  |  |  |  |  |  |  |  |  |  |  |
|  |  |  | Liquiritin | 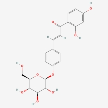 |  |  |  |  |  |  |
|  |  |  |  |  |  |  |  |  |  |  |
|  |  |  |  |  |  |  |  |  |  |  |
|  |  |  |  |  |  |  |  |  |  |  |
|  |  |  |  |  |  |  |  |  |  |  |
|  |  |  | Benzoic Acid | 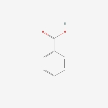 |  |  |  |  |  |  |
|  |  |  |  |  |  |  |  |  |  |  |
|  |  |  |  |  |  |  |  |  |  |  |
|  |  |  |  |  |  |  |  |  |  |  |
|  |  |  |  |  |  |  |  |  |  |  |
|  |  |  | Isoliquiritin apioside | 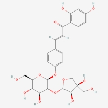 |  |  |  |  |  |  |
|  |  |  |  |  |  |  |  |  |  |  |
|  |  |  |  |  |  |  |  |  |  |  |
|  |  |  |  |  |  |  |  |  |  |  |
|  |  |  | Isoliquiritin | 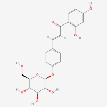 |  |  |  |  |  |  |
|  |  |  |  |  |  |  |  |  |  |  |
|  |  |  |  |  |  |  |  |  |  |  |
|  |  |  |  |  |  |  |  |  |  |  |
|  |  |  | Liquiritigenin |  |  |  |  |  |  |  |
|  |  |  |  |  |  |  |  |  |  |  |
|  |  |  |  |  |  |  |  |  |  |  |
|  |  |  |  |  |  |  |  |  |  |  |
|  |  |  | Glycyrrhizin |  |  |  |  |  |  |  |
|  |  |  |  |  |  |  |  |  |  |  |
|  |  |  |  |  |  |  |  |  |  |  |
|  |  |  |  |  |  |  |  |  |  |  |
|  |  |  | Trihydroxychalcone |  |  |  |  |  |  |  |
|  |  |  |  |  |  |  |  |  |  |  |
|  |  |  |  |  |  |  |  |  |  |  |
|  |  |  |  |  |  |  |  |  |  |  |
|  |  |  | Enoxolone |  |  |  |  |  |  |  |
|  |  |  |  |  |  |  |  |  |  |  |
|  |  |  |  |  |  |  |  |  |  |  |
|  |  |  |  |  |  |  |  |  |  |  |
|  |  |  |  |  |  |  |  |  |  |  |
|  | Tingli Dazao Xiefei Decoction | *Descurainia* Sophia (L.) Webb. et Prantl. [ Cruciferae, Descurainiae Semen ], ZiziPhus jujube Mill. [ Rhamnaceae, Fructus Zizyphi Jujubae ] | 6-O-alpha-D-mannopyranosyl-alpha-D-mannopyranose |  | Asthma-intraperitoneally injected [10mg/mL OVA and 10mg/mL AL(OH)3] and nebulized inhalation 2% OVA | Lachnospiraceae_NK4A136↑, Prevotellaceae_Ga6A1↑, Ruminococaceae UCG_005↑, Intestinimonas↑, Colidextribacter↑ | - | Symptoms of asthmatic behaviour↓; Infiltration of bronchial inflammatory cells↓; Lung and colon:iNOS↓, P–NF-κB-P65↓, IL-1β↓, CD4 + T cells/CD8 + T cells↓, TNF-α↓, IL-1α↓, IL-6↓ | Tingli Dazao Xiefei Decoction can enhance NO-CO metabolism in the lungs, which in turn benefits NO-CO metabolism in the intestines. This leads to coordinated regulation of inflammation, immune imbalance, cell barrier damage, oxidative stress, and gut bacteria in both the lungs and intestines, addressing asthma in vivo and in vitro. Targeting NO-CO metabolic disorders in these organs could be a novel approach for asthma treatment. | (Chen et al., 2023; Ruan et al., 2023) |
|  |  |  |  |  |  |  |  |  |  |  |
|  |  |  |  |  |  |  |  |  |  |  |
|  |  |  |  |  |  |  |  |  |  |  |
|  |  |  | Lewis a Trisaccharide |  |  |  |  |  |  |  |
|  |  |  |  |  |  |  |  |  |  |  |
|  |  |  |  |  |  |  |  |  |  |  |
|  |  |  |  |  |  |  |  |  |  |  |
|  |  |  | Glucoiberin |  |  |  |  |  |  |  |
|  |  |  |  |  |  |  |  |  |  |  |
|  |  |  |  |  |  |  |  |  |  |  |
|  |  |  |  |  |  |  |  |  |  |  |
|  |  |  |  |  |  |  |  |  |  |  |
|  |  |  | Guanine |  |  |  |  |  |  |  |
|  |  |  |  |  |  |  |  |  |  |  |
|  |  |  |  |  |  |  |  |  |  |  |
|  |  |  |  |  |  |  |  |  |  |  |
|  |  |  | Glucocheirolin |  |  |  |  |  |  |  |
|  |  |  |  |  |  |  |  |  |  |  |
|  |  |  |  |  |  |  |  |  |  |  |
|  |  |  |  |  |  |  |  |  |  |  |
|  |  |  | Glucoraphanin |  |  |  |  |  |  |  |
|  |  |  |  |  |  |  |  |  |  |  |
|  |  |  |  |  |  |  |  |  |  |  |
|  |  |  |  |  |  |  |  |  |  |  |
|  |  |  | Sinigrin |  |  |  |  |  |  |  |
|  |  |  |  |  |  |  |  |  |  |  |
|  |  |  |  |  |  |  |  |  |  |  |
|  |  |  |  |  |  |  |  |  |  |  |
|  |  |  | Gluconapin |  |  |  |  |  |  |  |
|  |  |  |  |  |  |  |  |  |  |  |
|  |  |  |  |  |  |  |  |  |  |  |
|  |  |  |  |  |  |  |  |  |  |  |
|  |  |  |  |  |  |  |  |  |  |  |
|  |  |  | Uracil |  |  |  |  |  |  |  |
|  |  |  |  |  |  |  |  |  |  |  |
|  |  |  |  |  |  |  |  |  |  |  |
|  |  |  |  |  |  |  |  |  |  |  |
|  |  |  | Glucosinalbin |  |  |  |  |  |  |  |
|  |  |  |  |  |  |  |  |  |  |  |
|  |  |  |  |  |  |  |  |  |  |  |
|  |  |  |  |  |  |  |  |  |  |  |
|  |  |  |  |  |  |  |  |  |  |  |
|  |  |  | Glucobrassicanapin |  |  |  |  |  |  |  |
|  |  |  |  |  |  |  |  |  |  |  |
|  |  |  |  |  |  |  |  |  |  |  |
|  |  |  |  |  |  |  |  |  |  |  |
|  |  |  |  |  |  |  |  |  |  |  |
|  |  |  | Glucoerucin |  |  |  |  |  |  |  |
|  |  |  |  |  |  |  |  |  |  |  |
|  |  |  |  |  |  |  |  |  |  |  |
|  |  |  |  |  |  |  |  |  |  |  |
|  |  |  |  |  |  |  |  |  |  |  |
|  | *Allium tuberosum* Rottler ex Sprengle | Liliaceae, Allium tuberosum | 1-O-feruloyl-beta-D-glucose |  | Asthma-intraperitoneally injected [20μg OVA and 100μL AL(OH)3] and nebulized inhalation 1% OVA | Firmicutes / Bacteroidetes(F/B)↑; Ruminococcus↑, Desulfovibrionaceae↑ | - | Inflammatory cell infiltration in the lung tissue↓, Mucus secretion in the lung tissue↓; Lung and gut:ILCs↓, blood serum :IgE↓; Eosinophils in BALF↓ | Allium tuberosum Rottler ex Sprengle mitigates pulmonary inflammation in murine models of asthma by suppressing the activation of innate lymphoid cells (ILCs) and modulating the homeostasis of the gut microbiota. | (Tang et al., 2017; Zheng et al., 2021) |
|  |  |  |  |  |  |  |  |  |  |  |
|  |  |  |  |  |  |  |  |  |  |  |
|  |  |  |  |  |  |  |  |  |  |  |
|  |  |  | Tachioside |  |  |  |  |  |  |  |
|  |  |  |  |  |  |  |  |  |  |  |
|  |  |  |  |  |  |  |  |  |  |  |
|  |  |  |  |  |  |  |  |  |  |  |
|  |  |  |  |  |  |  |  |  |  |  |
|  |  |  | Kaempferol 3-O-sophoroside |  |  |  |  |  |  |  |
|  |  |  |  |  |  |  |  |  |  |  |
|  |  |  |  |  |  |  |  |  |  |  |
|  |  |  |  |  |  |  |  |  |  |  |
|  |  |  | Urolignoside |  |  |  |  |  |  |  |
|  |  |  |  |  |  |  |  |  |  |  |
|  |  |  |  |  |  |  |  |  |  |  |
|  |  |  |  |  |  |  |  |  |  |  |
|  |  |  |  |  |  |  |  |  |  |  |
|  |  |  | Kaempferol 3,4'-diglucoside |  |  |  |  |  |  |  |
|  |  |  |  |  |  |  |  |  |  |  |
|  |  |  |  |  |  |  |  |  |  |  |
|  |  |  |  |  |  |  |  |  |  |  |
|  |  |  | Kaempferol-3-O-rutinoside |  |  |  |  |  |  |  |
|  |  |  |  |  |  |  |  |  |  |  |
|  |  |  |  |  |  |  |  |  |  |  |
|  |  |  |  |  |  |  |  |  |  |  |
|  | Eriobotrya japonica (Thunb.) Lindl. | Rosaceae, Eriobotryae Folium | Oleanolic Acid |  | Cough Variant Asthma-intraperitoneally injected (50μg OVA and alum) and nebulized inhalation 1% OVA | Bacteroidetes↓, Euryarchaeota↓, Lactobacillus↑; Methanobrevibacter↓, Prevotellaceae UCG-003↓, Rikenellaceae_RC9_gut_group↓, escherichia↓, Prevotellaceae_ga6a1_group↓, clostridium↓, Bactoscilla↓, Alloprevotella↓ | - | Behavioral symptoms of asthma↓, Inflammatory cell infiltration in the lung tissue↓, Airway wall thickness↓, α-SMA↓, MMP-9↓, TIMP-1↓ | Eriobotrya japonica (Thunb.) Lindl. treats cough variant asthma (CVA) through traditional Chinese medicine principles, focusing on airway remodeling, intestinal flora, and the MMP-9/TIMP-1 pathway. Results indicated that EJL reduces cough frequency, extends cough latency, improves body weight, and positively impacts lung tissue pathology. | (He et al., 2021) |
|  |  |  |  |  |  |  |  |  |  |  |
|  |  |  |  |  |  |  |  |  |  |  |
|  |  |  |  |  |  |  |  |  |  |  |
|  |  |  | Ursolic Acid |  |  |  |  |  |  |  |
|  |  |  |  |  |  |  |  |  |  |  |
|  |  |  |  |  |  |  |  |  |  |  |
|  |  |  |  |  |  |  |  |  |  |  |
|  |  |  |  |  |  |  |  |  |  |  |
|  | *Hylocereus undatus* (Haw.) Britt. et Rose | Cactaceae, Hylocere usundatus | L-Arabinose |  | Asthma-intraperitoneally injected [20μg OVA and 2mg AL(OH)3] and nebulized inhalation 1% OVA | Firmicutes / Bacteroidetes(F/B)↑, Lachnospiraceae UCG-001↑, Eubacterium_xylanophilum_group↑, Muribaculum↑, Rikenella↑, Oscillibacter↑, Lachnospiraceae_FCS020_group↑, Lachnospiraceae_NK4A136_group↑, Roseburia_decreased↑, Muribaculaceae↓, Alloprevotella↓ | - | Symptoms of asthmatic behaviour↓, BALF: eosinophils↓, neutrophile granulocyte ↓, lymphocytoponia↓; Inflammatory cell infiltration in the lung tissue and around the bronchus↓; blood serum :IgE↓, IL-4↓, IL-5↓, IL-13↓, IL-1β↓, TNF-α↓, p-p38 MAPK↓, p-NF-κB p65↓, SIRT1↑, caspase-1 ↓; Histopathological damage of the colon↓; colon:IL-1β↓, caspase-1↓, SIRT1↑ | Hylocereus undatus (Haw.) Britt. et Rose alleviated asthma symptoms by decreasing respiratory rate, inflammatory cell buildup, airway inflammation, mucus secretion, and lung collagen deposition. It also improved the gut environment in asthmatic mice by modulating lung and intestinal microflora, boosting SIRT1 protein expression, and inhibiting p38MAPK, NF-κBp65, and caspase-1 protein activities. | (Yi et al., 2012; Liao et al., 2022) |
|  |  |  |  |  |  |  |  |  |  |  |
|  |  |  |  |  |  |  |  |  |  |  |
|  |  |  |  |  |  |  |  |  |  |  |
|  |  |  | D-Galactose |  |  |  |  |  |  |  |
|  |  |  |  |  |  |  |  |  |  |  |
|  |  |  |  |  |  |  |  |  |  |  |
|  |  |  |  |  |  |  |  |  |  |  |
|  |  |  |  |  |  |  |  |  |  |  |
|  |  |  | L-Fucose |  |  |  |  |  |  |  |
|  |  |  |  |  |  |  |  |  |  |  |
|  |  |  |  |  |  |  |  |  |  |  |
|  |  |  |  |  |  |  |  |  |  |  |
|  |  |  |  |  |  |  |  |  |  |  |
|  |  |  | Biorobin |  |  |  |  |  |  |  |
|  |  |  |  |  |  |  |  |  |  |  |
|  |  |  |  |  |  |  |  |  |  |  |
|  |  |  |  |  |  |  |  |  |  |  |
|  |  |  |  |  |  |  |  |  |  |  |
|  |  |  | Kaempferol-3-O-rutinoside |  |  |  |  |  |  |  |
|  |  |  |  |  |  |  |  |  |  |  |
|  |  |  |  |  |  |  |  |  |  |  |
|  |  |  |  |  |  |  |  |  |  |  |
|  |  |  |  |  |  |  |  |  |  |  |
|  |  |  | Isorhamnetin 3-O-robinobioside |  |  |  |  |  |  |  |
|  |  |  |  |  |  |  |  |  |  |  |
|  |  |  |  |  |  |  |  |  |  |  |
|  |  |  |  |  |  |  |  |  |  |  |
|  |  |  |  |  |  |  |  |  |  |  |
|  |  |  | Narcissin |  |  |  |  |  |  |  |
|  |  |  |  |  |  |  |  |  |  |  |
|  |  |  |  |  |  |  |  |  |  |  |
|  |  |  |  |  |  |  |  |  |  |  |
|  |  |  | Astragalin |  |  |  |  |  |  |  |
|  |  |  |  |  |  |  |  |  |  |  |
|  |  |  |  |  |  |  |  |  |  |  |
|  |  |  |  |  |  |  |  |  |  |  |
|  |  |  | Isorhamnetin 3-O-glucoside |  |  |  |  |  |  |  |
|  |  |  |  |  |  |  |  |  |  |  |
|  |  |  |  |  |  |  |  |  |  |  |
|  |  |  |  |  |  |  |  |  |  |  |
|  | Lycium barbarumpolysaccharide | Solanaceae, Lycii Fructus | D-Mannose |  | Allergic Asthma-intraperitoneally injected [100μg OVA and 4mg AL(OH)3] and intranasal administration 50μL OVA | Firmicutes↓, Actinomyces↓, Alistipes↓, Lactobacillus↑, Bacillus bifidus ↑, Clostridiales↑ | - | Behavioral symptoms of asthma↓, Infiltration of lymphocytes and mast cells in and around the airways↓; BALF and blood plasma:TNF↓, MCP-1↓, IL-6↓; IL-4↓IL-17A↓; airway remodeling↓ | Lycium barbarumpolysaccharide may alleviate lung injury and inflammation in mice, as well as alter gut microbiota by increasing lactic acid bacteria and bifidobacteria while reducing Firmicutes and Actinobacteria. Lycium barbarumpolysaccharide could improve allergic asthma by modulating gut microbiota and reducing inflammation. | [(](#_ENREF_20) (Tang et al., 2015; Cui et al., 2020) |
|  |  |  |  |  |  |  |  |  |  |  |
|  |  |  |  |  |  |  |  |  |  |  |
|  |  |  |  |  |  |  |  |  |  |  |
|  |  |  |  |  |  |  |  |  |  |  |
|  |  |  | L-Rhamnose |  |  |  |  |  |  |  |
|  |  |  |  |  |  |  |  |  |  |  |
|  |  |  |  |  |  |  |  |  |  |  |
|  |  |  |  |  |  |  |  |  |  |  |
|  |  |  |  |  |  |  |  |  |  |  |
|  |  |  | D-Glucose |  |  |  |  |  |  |  |
|  |  |  |  |  |  |  |  |  |  |  |
|  |  |  |  |  |  |  |  |  |  |  |
|  |  |  |  |  |  |  |  |  |  |  |
|  |  |  | D-Galactose |  |  |  |  |  |  |  |
|  |  |  |  |  |  |  |  |  |  |  |
|  |  |  |  |  |  |  |  |  |  |  |
|  |  |  |  |  |  |  |  |  |  |  |
|  |  |  | D-Xylose |  |  |  |  |  |  |  |
|  |  |  |  |  |  |  |  |  |  |  |
|  |  |  |  |  |  |  |  |  |  |  |
|  |  |  |  |  |  |  |  |  |  |  |
|  | Tetrahydrocurcumin | Zingiberaceae , Curcumae Longae Rhizoma | Tetrahydrocurcumin |  | Allergic Asthma-intraperitoneally injected [40μg OVA and 2mg AL(OH)3] and nebulized inhalation 5% OVA | Firmicutes / Bacteroidetes(F/B)↑; Ruminococcus ↓, Parabacteroides ↓, Intestinimonas ↓ | - | Behavioral symptoms of asthma↓, Eosinophil infiltration in the lung tissue↓; lung : Th2↓, Th17↓, Tc2↓, Tc17↓; Histopathological damage of the colon↓ | In the context of allergic asthma in murine models, it was observed that Tetrahydrocurcumin mitigated allergic airway inflammation through the modulation of intestinal microbiota. This modulation subsequently alleviated symptoms and reduced Th2-mediated inflammation in both pulmonary and colonic tissues. | (Wu et al., 2021b; Qin et al., 2023) |
|  |  |  |  |  |  |  |  |  |  |  |
|  |  |  |  |  |  |  |  |  |  |  |
|  |  |  |  |  |  |  |  |  |  |  |

**Table 1 References**

Chen, H., Zhu, Y., Zhao, X., and Yang, Z. (2023). Tingli Dazao Decoction pretreatment ameliorates mitochondrial damage induced by oxidative stress in cardiomyocytes. J Ethnopharmacol 303, 115987. doi: [10.1016/j.jep.2022.115987](https://doi.org/10.1016/j.jep.2022.115987)

Cui, F., Shi, C.-L., Zhou, X.-J., Wen, W., Gao, X.-P., Wang, L.-Y., et al. (2020). Lycium barbarum Polysaccharide Extracted from Lycium barbarum Leaves Ameliorates Asthma in Mice by Reducing Inflammation and Modulating Gut Microbiota. J Med Food 23, 699–710. doi: [10.1089/jmf.2019.4544](https://doi.org/10.1089/jmf.2019.4544)

Dai, X., Liu, Y., Liu, T., Zhang, Y., Wang, S., Xu, T., et al. (2024). SiJunZi decoction ameliorates bone quality and redox homeostasis and regulates advanced glycation end products/receptor for advanced glycation end products and WNT/β-catenin signaling pathways in diabetic mice. J Ethnopharmacol 319, 117167. doi: [10.1016/j.jep.2023.117167](https://doi.org/10.1016/j.jep.2023.117167)

Dong, Y., Hu, S., Liu, C., Meng, Q., Song, J., Lu, J., et al. (2015). Purification of polysaccharides from Cordyceps militaris and their anti‑hypoxic effect. Mol Med Rep 11, 1312–1317. doi: [10.3892/mmr.2014.2786](https://doi.org/10.3892/mmr.2014.2786)

Dong, Y., Yan, H., Zhao, X., Lin, R., Lin, L., Ding, Y., et al. (2020). Gu-Ben-Fang-Xiao Decoction Ameliorated Murine Asthma in Remission Stage by Modulating Microbiota-Acetate-Tregs Axis. Front Pharmacol 11, 549. doi: [10.3389/fphar.2020.00549](https://doi.org/10.3389/fphar.2020.00549)

H, H., G, Z., K, W., P, H., H, Y., F, W., et al. (2023). Study on the Mechanism of Qing-Fei-Shen-Shi Decoction on Asthma Based on Integrated 16S rRNA Sequencing and Untargeted Metabolomics. Evidence-based complementary and alternative medicine : eCAM 2023. doi: [10.1155/2023/1456844](https://doi.org/10.1155/2023/1456844)

He, Q., Liu, C., Shen, L., Zeng, L., Wang, T., Sun, J., et al. (2021). Theory of the exterior-interior relationship between the lungs and the large intestine to explore the mechanism of Eriobotrya japonica leaf water extract in the treatment of cough variant asthma. J Ethnopharmacol 281, 114482. doi: [10.1016/j.jep.2021.114482](https://doi.org/10.1016/j.jep.2021.114482)

He, R., Wang, S., Yang, S., Liu, R., Nan, N., Lu, X., et al. (2023). Shaoyao-Gancao-Tang regulates the T-helper-type 1/T-helper-type 2 ratio in the lung and gut and alters gut microbiota in rats with ovalbumin-induced asthma. J Ethnopharmacol 309, 116300. doi: [10.1016/j.jep.2023.116300](https://doi.org/10.1016/j.jep.2023.116300)

Hsu, W.-H., Lin, L.-J., Lu, C.-K., Kao, S.-T., and Lin, Y.-L. (2021). Effect of You-Gui-Wan on House Dust Mite-Induced Mouse Allergic Asthma via Regulating Amino Acid Metabolic Disorder and Gut Dysbiosis. Biomolecules 11, 812. doi: [10.3390/biom11060812](https://doi.org/10.3390/biom11060812)

Jia, W., Xu, C., Zhao, T., Fan, Q., Qiao, B., Wu, Y., et al. (2023). Integrated Network Pharmacology and Gut Microbiota Analysis to Explore the Mechanism of Sijunzi Decoction Involved in Alleviating Airway Inflammation in a Mouse Model of Asthma. Evid Based Complement Alternat Med 2023, 1130893. doi: [10.1155/2023/1130893](https://doi.org/10.1155/2023/1130893)

Jx, L., Hy, Y., Yn, L., Z, W., Y, L., and J, L. (2022). Ephedra sinica polysaccharide alleviates airway inflammations of mouse asthma-like induced by PM2.5 and ovalbumin via the regulation of gut microbiota and short chain fatty acid. The Journal of pharmacy and pharmacology 74. doi: [10.1093/jpp/rgac078](https://doi.org/10.1093/jpp/rgac078)

Kong, Y. H., Shi, Q., Han, N., Zhang, L., Zhang, Y. Y., Gao, T. X., et al. (2016). Structural Modulation of Gut Microbiota in Rats with Allergic Bronchial Asthma Treated with Recuperating Lung Decoction. Biomed Environ Sci 29, 574–583. doi: [10.3967/bes2016.076](https://doi.org/10.3967/bes2016.076)

Liao, W., Liu, W., Yan, Y., Li, L., Tong, J., Huang, Y., et al. (2022). Hylocereus undatus flower extract suppresses OVA-induced allergic asthma in BALb/c mice by reducing airway inflammation and modulating gut microbiota. Biomed Pharmacother 153, 113476. doi: [10.1016/j.biopha.2022.113476](https://doi.org/10.1016/j.biopha.2022.113476)

Liu, F., Duan, W., Guan, T., Zhou, Q., Yan, W., and Geng, Y. (2023a). Water extract of Pingchuan formula ameliorated murine asthma through modulating metabolites and gut microbiota. J Pharm Biomed Anal 236, 115728. doi: [10.1016/j.jpba.2023.115728](https://doi.org/10.1016/j.jpba.2023.115728)

Liu, Z., Li, Y., Li, N., Wang, Y., Li, Q., Ge, D., et al. (2023b). Dachengqi Decoction alleviates intestinal inflammation in ovalbumin-induced asthma by reducing group 2 innate lymphoid cells in a microbiota-dependent manner. J Tradit Complement Med 13, 183–192. doi: [10.1016/j.jtcme.2023.01.003](https://doi.org/10.1016/j.jtcme.2023.01.003)

Lu, Y., Xu, J.-Y., Zhang, X.-H., and Zhao, X. (2016). Gu-Ben-Fang-Xiao decoction attenuates sustained airway inflammation by suppressing ER stress response in a murine asthma remission model of respiratory syncytial virus infection. J Ethnopharmacol 192, 496–509. doi: [10.1016/j.jep.2016.09.039](https://doi.org/10.1016/j.jep.2016.09.039)

Qin, J., Lv, M., Jiang, Z., Meng, X., Wang, Y., Cui, J., et al. (2021). Tuo-Min-Ding-Chuan Decoction Alleviate Ovalbumin-Induced Allergic Asthma by Inhibiting Mast Cell Degranulation and Down-Regulating the Differential Expression Proteins. Front Pharmacol 12, 725953. doi: [10.3389/fphar.2021.725953](https://doi.org/10.3389/fphar.2021.725953)

Qin, Y.-Q., Wang, L.-Y., Yang, X.-Y., Xu, Y.-J., Fan, G., Fan, Y.-G., et al. (2023). Inulin: properties and health benefits. Food Funct 14, 2948–2968. doi: [10.1039/d2fo01096h](https://doi.org/10.1039/d2fo01096h)

Ruan, Y., Yuan, P.-P., Li, P.-Y., Chen, Y., Fu, Y., Gao, L.-Y., et al. (2023). Tingli Dazao Xiefei Decoction ameliorates asthma in vivo and in vitro from lung to intestine by modifying NO-CO metabolic disorder mediated inflammation, immune imbalance, cellular barrier damage, oxidative stress and intestinal bacterial disorders. J Ethnopharmacol 313, 116503. doi: [10.1016/j.jep.2023.116503](https://doi.org/10.1016/j.jep.2023.116503)

Song, L., Yang, J., Kong, W., Liu, Y., Liu, S., and Su, L. (2023). Cordyceps militaris polysaccharide alleviates ovalbumin-induced allergic asthma through the Nrf2/HO-1 and NF-κB signaling pathways and regulates the gut microbiota. Int J Biol Macromol 238, 124333. doi: [10.1016/j.ijbiomac.2023.124333](https://doi.org/10.1016/j.ijbiomac.2023.124333)

Soua, L., Koubaa, M., Barba, F. J., Fakhfakh, J., Ghamgui, H. K., and Chaabouni, S. E. (2020). Water-Soluble Polysaccharides from Ephedra alata Stems: Structural Characterization, Functional Properties, and Antioxidant Activity. Molecules 25, 2210. doi: [10.3390/molecules25092210](https://doi.org/10.3390/molecules25092210)

Tang, D., Wang, C., Gan, Q., Wang, Z., and Jiang, R. (2022). Chemical composition-based characterization of the anti-allergic effect of Guominkang Formula on IgE-mediated mast cells activation and passive cutaneous anaphylaxis. Chin J Nat Med 20, 925–936. doi: [10.1016/S1875-5364(22)60225-5](https://doi.org/10.1016/S1875-5364(22)60225-5)

Tang, H.-L., Chen, C., Wang, S.-K., and Sun, G.-J. (2015). Biochemical analysis and hypoglycemic activity of a polysaccharide isolated from the fruit of Lycium barbarum L. Int J Biol Macromol 77, 235–242. doi: [10.1016/j.ijbiomac.2015.03.026](https://doi.org/10.1016/j.ijbiomac.2015.03.026)

Tang, X., Olatunji, O. J., Zhou, Y., and Hou, X. (2017). Allium tuberosum: Antidiabetic and hepatoprotective activities. Food Res Int 102, 681–689. doi: [10.1016/j.foodres.2017.08.034](https://doi.org/10.1016/j.foodres.2017.08.034)

Tsang, M. S. M., Jiao, D., Chan, B. C. L., Hon, K.-L., Leung, P. C., Lau, C. B. S., et al. (2016). Anti-Inflammatory Activities of Pentaherbs Formula, Berberine, Gallic Acid and Chlorogenic Acid in Atopic Dermatitis-Like Skin Inflammation. Molecules 21, 519. doi: [10.3390/molecules21040519](https://doi.org/10.3390/molecules21040519)

Tsang, M. S.-M., Cheng, S.-W., Zhu, J., Atli, K., Chan, B. C.-L., Liu, D., et al. (2018). Anti-Inflammatory Activities of Pentaherbs formula and Its Influence on Gut Microbiota in Allergic Asthma. Molecules 23, 2776. doi: [10.3390/molecules23112776](https://doi.org/10.3390/molecules23112776)

Wang, L., Cao, A.-L., Chi, Y.-F., Ju, Z.-C., Yin, P.-H., Zhang, X.-M., et al. (2015). You-gui Pill ameliorates renal tubulointerstitial fibrosis via inhibition of TGF-β/Smad signaling pathway. J Ethnopharmacol 169, 229–238. doi: [10.1016/j.jep.2015.04.037](https://doi.org/10.1016/j.jep.2015.04.037)

Wu, Y., Chen, Y., Li, Q., Ye, X., Guo, X., Sun, L., et al. (2021). Tetrahydrocurcumin alleviates allergic airway inflammation in asthmatic mice by modulating the gut microbiota. Food Funct 12, 6830–6840. doi: [10.1039/d1fo00194a](https://doi.org/10.1039/d1fo00194a)

Yi, Y., Zhang, Q.-W., Li, S.-L., Wang, Y., Ye, W.-C., Zhao, J., et al. (2012). Simultaneous quantification of major flavonoids in “Bawanghua”, the edible flower of Hylocereus undatus using pressurised liquid extraction and high performance liquid chromatography. Food Chem 135, 528–533. doi: [10.1016/j.foodchem.2012.05.010](https://doi.org/10.1016/j.foodchem.2012.05.010)

Yin, F.-T., Zhou, X.-H., Kang, S.-Y., Li, X.-H., Li, J., Ullah, I., et al. (2022). Prediction of the mechanism of Dachengqi Decoction treating colorectal cancer based on the analysis method of “ into serum components -action target-key pathway.” J Ethnopharmacol 293, 115286. doi: [10.1016/j.jep.2022.115286](https://doi.org/10.1016/j.jep.2022.115286)

Yuan, G., Wen, S., Zhong, X., Yang, X., Xie, L., Wu, X., et al. (2023). Inulin alleviates offspring asthma by altering maternal intestinal microbiome composition to increase short-chain fatty acids. PLoS One 18, e0283105. doi: [10.1371/journal.pone.0283105](https://doi.org/10.1371/journal.pone.0283105)

Zheng, H.-C., Liu, Z.-R., Li, Y.-L., Wang, Y.-A., Kong, J.-W., Ge, D.-Y., et al. (2021). Allium tuberosum alleviates pulmonary inflammation by inhibiting activation of innate lymphoid cells and modulating intestinal microbiota in asthmatic mice. J Integr Med 19, 158–166. doi: [10.1016/j.joim.2020.11.003](https://doi.org/10.1016/j.joim.2020.11.003)

Zhou, Y., Hu, L., Zhang, H., Zhang, H., Liu, J., Zhao, X., et al. (2022a). Guominkang formula alleviate inflammation in eosinophilic asthma by regulating immune balance of Th1/2 and Treg/Th17 cells. Front Pharmacol 13, 978421. doi: [10.3389/fphar.2022.978421](https://doi.org/10.3389/fphar.2022.978421)

Zhou, Y., Zhao, H., Wang, T., Zhao, X., Wang, J., and Wang, Q. (2022b). Anti-Inflammatory and Anti-asthmatic Effects of TMDCT Decoction in Eosinophilic Asthma Through Treg/Th17 Balance. Front Pharmacol 13, 819728. doi: [10.3389/fphar.2022.819728](https://doi.org/10.3389/fphar.2022.819728)
